# Analysis on evolutionary relationship of amylases from archaea, bacteria and eukaryota

**DOI:** 10.1007/s11274-015-1979-y

**Published:** 2016-01-08

**Authors:** Shaomin Yan, Guang Wu

**Affiliations:** State Key Laboratory of Non-food Biomass Enzyme Technology, National Engineering Research Center for Non-food Biorefinery, Guangxi Biomass Industrialization Engineering Institute, Guangxi Key Laboratory of Biorefinery, Guangxi Academy of Sciences, 98 Daling Road, Nanning, 530007 Guangxi China

**Keywords:** Amylase, Engineering, Evolution, Phylogenetics, Statistics

## Abstract

Amylase is one of the earliest characterized enzymes and has many applications in clinical and industrial settings. In biotechnological industries, the amylase activity is enhanced through modifying amylase structure and through cloning and expressing targeted amylases in different species. It is important to understand how engineered amylases can survive from generation to generation. This study used phylogenetic and statistical approaches to explore general patterns of amylases evolution, including 3118 α-amylases and 280 β-amylases from archaea, eukaryota and bacteria with fully documented taxonomic lineage. First, the phylogenetic tree was created to analyze the evolution of amylases with focus on individual amylases used in biofuel industry. Second, the average pairwise *p*-distance was computed for each kingdom, phylum, class, order, family and genus, and its diversity implies multi-time and multi-clan evolution. Finally, the variance was further partitioned into inter-clan variance and intra-clan variance for each taxonomic group, and they represent horizontal and vertical gene transfer. Theoretically, the results show a full picture on the evolution of amylases in manners of vertical and horizontal gene transfer, and multi-time and multi-clan evolution as well. Practically, this study provides the information on the surviving chance of desired amylase in a given taxonomic group, which may potentially enhance the successful rate of cloning and expression of amylase gene in different species.

**Electronic supplementary material:**

The online version of this article (doi:10.1007/s11274-015-1979-y) contains supplementary material, which is available to authorized users.

## Introduction

Amylase catalyzes the hydrolysis of 1,4-glycosidic linkages of starch to sugar. So far, several groups of amylases have been defined (Schomburg et al. 2013), and α- and β-amylases have many applications in clinical and biotechnological settings.

α-Amylase, EC 3.2.1.1, is generally considered to account for the majority of amylases in nature, and is the major component in various databases. There are 3398 amylases with fully documented taxonomic lineage in the release 2014_02 of UniProtKB (The UniProt Consortium 2010), and 3118 (91.76 %) are α-amylases. α-Amylase widely exists across taxonomic kingdoms, for these 3398 amylases, α-amylases account for 100, 98.47 and 66.99 % of amylases in archaea, bacteria and eukaryota, respectively. α-Amylase has wide applications in both clinical and industrial settings. In clinical settings, great interest is directed to hyperamylasemia that is associated with pancreatitis (Elsayed et al. 2014; Hernández Garcés et al. 2014) and biliopancreatic duct ligation (Fishman et al. 2014), and the α-amylase level in urine is also clinically meaningful (Voskuil et al. 2014). Still, α-amylase serves as a predictor of pancreatic fistula (Partelli et al. 2014), and a salivary biomarker for acute stress (Koh 2014), gastricaspiration (Sole et al. 2014), etc. On the one hand, α-amylase plays a role in degradation of dietary components by digestive tract microbiotic flora (Tzuc et al. 2014). On the other hand, inhibition of α-amylase is equally important for the control of post-prandial hyperglycemia (Wulan et al. 2014), so the inhibition of human salivary α-amylase could be potentially useful in the prevention and treatment of obesity and type 2 diabetes as well (Bharathi et al. 2014; Gupta et al. 2014; Podsędek et al. 2014; Tiwari 2014). In addition, applications of α-amylase are widely used in fruit juice industry (Dey and Banerjee 2014), for example, bioconversion of wastes into hydrogen and methane (Kumar et al. 2014), bioethanol production (Pervez et al. 2014), textile industry (Deng et al. 2014), chicken feed enzyme (Jianhua et al. 2014), paper recycling (Raul et al. 2014), and detergent industry (Zaferanloo et al. 2014).

β-Amylase, EC 3.2.1.2, doubtlessly occupies the second position in various databases. Of 3398 amylases with fully documented taxonomic lineage in the release 2014_02 of UniProtKB, 280 (8.24 %) are β-amylases, which also widely exists across taxonomic kingdoms. For these 280 β-amylases, they account for 1.53 and 33.01 % of amylases in bacteria and eukaryota. β-Amylase works from the non-reducing end of starch and catalyzes the hydrolysis of the second α-1,4-glycosidic linkage to produce maltose (Peng et al. 2014). Branching enzyme alone and in combination with β-amylase were used to increase the amount of α-1,6 branching points, which are slowly hydrolyzed by mucosal α-glucosidases in the small intestine (Kittisuban et al. 2014).

As one of the earliest-found-enzymes, its evolution is always fascinating, not only because its roles are in clinical and industrial settings but also because a huge effort is being made in biotechnological industries to enhance amylase activity by modifying its structure. An interesting question is whether a human-modified amylase can survive with desired properties for generations in different species. An importance is whether a mutant can survive across species, i.e., when an amylase gene is cloned and expressed in *Escherichia coli*, can this foreign amylase gene function well in *E. coli*?

The analysis on the evolution of amylases can partially answer this question. Indeed, phylogenetics can reveal the evolutionary relationship entangled with human-modified amylases, and incorporation of large dataset can substantially enhance the revelation of phylogenetic analysis.

Additional approaches are still needed to answer this question. The first approach is to compute the average pairwise *p*-distance for each taxonomic group, which shows the diversity in each taxonomic group. A cloned and expressed amylase gene has a large chance to survive in a taxonomic group with a large average pairwise *p*-distance, because the previous observation showed that the larger the diversity is, the more the variants are (Darwin 1876). The second approach is to partition the variance into inter-clan and intra-clan variances with unequal size model II ANOVA, which tells whether a cloned and expressed amylases gene can easily survive in its own taxonomic group or in other taxonomic group. Theoretically, the average pairwise *p*-distance reveals the evolution in a multiple times manner and the partitioning of variance elucidates the horizontal gene transfer across species. It happens that these two approaches require the full taxonomic lineage from superkingdom, to kingdom, to phylum, to class, to order, to family, to genus and finally to species. Collectively, these three approaches can throw new insight into the evolution of amylases with respect to the abovementioned question.

## Materials and methods

### Data

When using amylase as a key searching word, two categories of amylases exist in UniProtKB, i.e., hydrolase and glycosidase. Thus, 4394 amylases under category of hydrolase and 4126 amylases under category of glycosidase were found in the UniProtKB. These 8520 amylases were all available amylases in UniProtKB for the release 2013_09—September 2013 and 2013_10—October 2013. After the deletion of identical amylases, 4721 amylases remained including 327 under the category of glycosidase, 852 under the category of hydrolase, and the rest 3542 under both categories of glycosidase and hydrolase.

The taxonomic lineage of the 4721 amylases was verified against the UniProtKB for release 2014_02. This verification found that 3398 amylases have fully documented taxonomic lineage from superkingdom, to kingdom, to phylum, to class, to order, to family, to genus, and finally to organism (Table S1 in Supplementary Materials). Hence, the 3398 amylases were used in this study. Of these 3398 amylases, 88 amylases come from archaea (Table S2 in Supplementary Materials) including two phyla (33 from *Crenarchaeota* and 55 from *Euryarchaeota*), seven classes (33 from *Thermoprotei*, 1 from *Archaeoglobi*, 14 from *Halobacteria*, 14 from *Methanococci*, 12 from *Methanomicrobia*, 13 from *Thermococci* and 1 from *Thermoplasmata*), 12 orders, 17 families and 35 genera; 724 amylases come from eukaryota (Table S2 in Supplementary Materials) including three kingdoms (53 from *Fungi*, 397 from *Metazoa* and 274 from *Viridiplantae*), 9 phyla, 23 classes, 46 orders, 83 families and 140 genera; 2586 amylases come from bacteria (Table S3 in Supplementary Materials) including 9 phyla, 18 classes, 41 orders, 67 families and 128 genera.

Of the 3398 amylases, 105 belong to the category of glycosidase, 359 belong to the category of hydrolase, 2934 belong to both categories of glycosidase and hydrolase, and 3118 are α-amylases and 280 are β-amylases. There are 88 α-amylases in archaea, 2545 α-amylases and 41 β-amylases in bacteria, and 485 α-amylases and 239 β-amylases in eukaryota.

### Phylogenetic analysis

The sequence alignment was carried out using ClustalX (Larkin et al. 2007), which is a version with graphical user interface in Clustal series for multiple sequence alignment. The ClustalX performs multiple sequence alignment through sequence weighting, position-specific gap penalties and weight matrix (Thompson et al. 1994). The phylogenetic tree was constructed using ClustalX with neighbor-joining method maximum likelihood, and presented using NJPlot (Perrière and Gouy 1996). The phylogenetic trees are statistically validated using 1000 bootstrap replicates in ClustalX. The default settings in ClustalX were chosen for operating ClustalX as recommended by software developers.

### Statistical analysis

The pairwise *p*-distance was computed using Mega version 6.06 (Tamura et al. 2013) for each superkingdom, kingdom, phylum, class, order, family and genus, and then was presented in heatmaps. Although Mega software has the function of multiple sequence alignment using Clustal algorithm, our experience shows that its performance is not as good as ClustalX in pairwise sequence alignment, so the aligned sequences were input into Mega for computation of pairwise *p*-distance, which is the proportion (*p*) of amino acid sites at which two sequences being compared are different.

The average pairwise *p*-distance was furthermore partitioned in terms of intra-clan (within-clan) variance and inter-clan (between-clan) variance using unequal size model II ANOVA (Sokal and Rohlf 1995; Wu et al. 1999; Yan and Wu 2009, 2010a, b, 2011, 2013a, b).

## Results

**Fig. 1:**
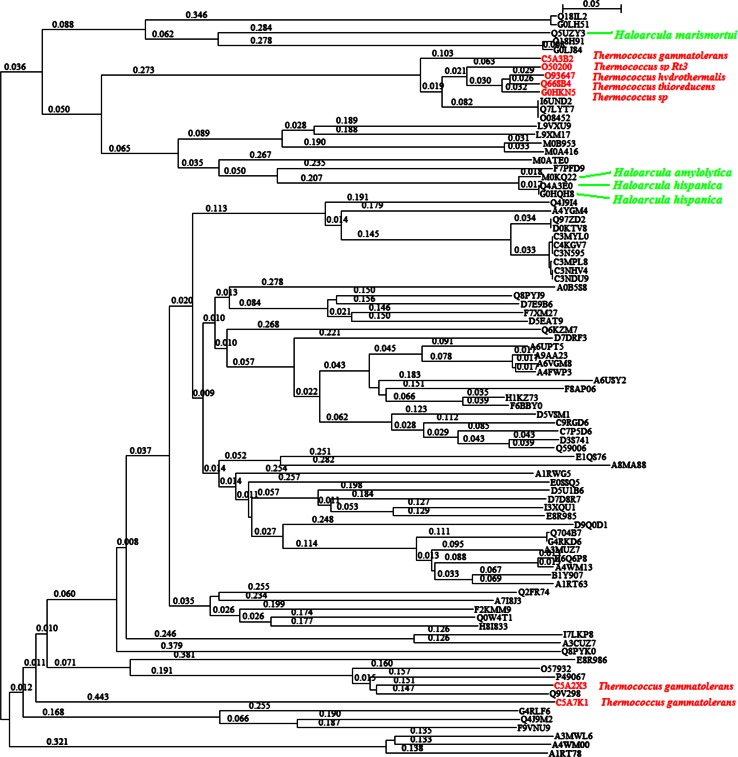
Phylogenetic tree of 88 α-amylases from archaea. The *numbers* along each branch and *scale bar* represent the branch length, which is the number of substitutions per unit time (Tamura et al. 2013). Seven α-amylases from genus *Thermococcus* were marked in *red* and four α-amylases from genus *Haloarcula* were marked in *green*

Figure 1 shows the phylogenetic tree of 88 α-amylases from archaea. These amylases represent just a very small portion of amylases in the database, but the interests in them strongly exist because several species from archaea can survive in extreme environments, which could be potentially useful in biotechnological industries. For example, seven α-amylases belong to genus *Thermococcus*, which are hyperthermophilic archaea (Jeon et al. 2014), marked with red in Fig. 1. As can be seen, they evolved into two clusters at two extremes of phylogenetic tree. Moreover, these seven α-amylases have experienced different steps to reach the branches in the phylogenetic tree, so it is highly likely that these two clusters evolved independently and separately. The quantification of these different steps can be associated with the branch length in Fig. 1. As a result, the hyperthermophilic property in archaea is unlikely to be inherited vertically because of their two extreme locations in phylogenetic tree. On the other hand, the evolution of hyperthermophilic property in archaea could be attributed to the extreme environments, which would be the natural selection (Darwin 1876) for two extreme locations in phylogenetic tree.

Another property is that haloarchaeal amylases have lower propensity for helix formation but higher propensity for coil formation, which keeps amylases being active in high salt concentration through highly negative charged surface with over representation of acidic residues (Liu et al. 2011; Zorgani et al. 2014). This haloadaptation in hypersaline environments can be tracked in Fig. 1 marked in green, where three amylases (G0HQH8, Q4A3E0 and M0KQ22) from *Haloarcula* clustered together whereas an amylase from *Haloarcula marismortui* evolved independently. However, two amylases from *Haloarcula hispanica* did evolve together, which is a piece of evidence of vertical inheritance for haloadaptation in archaea.

**Fig. 2:**
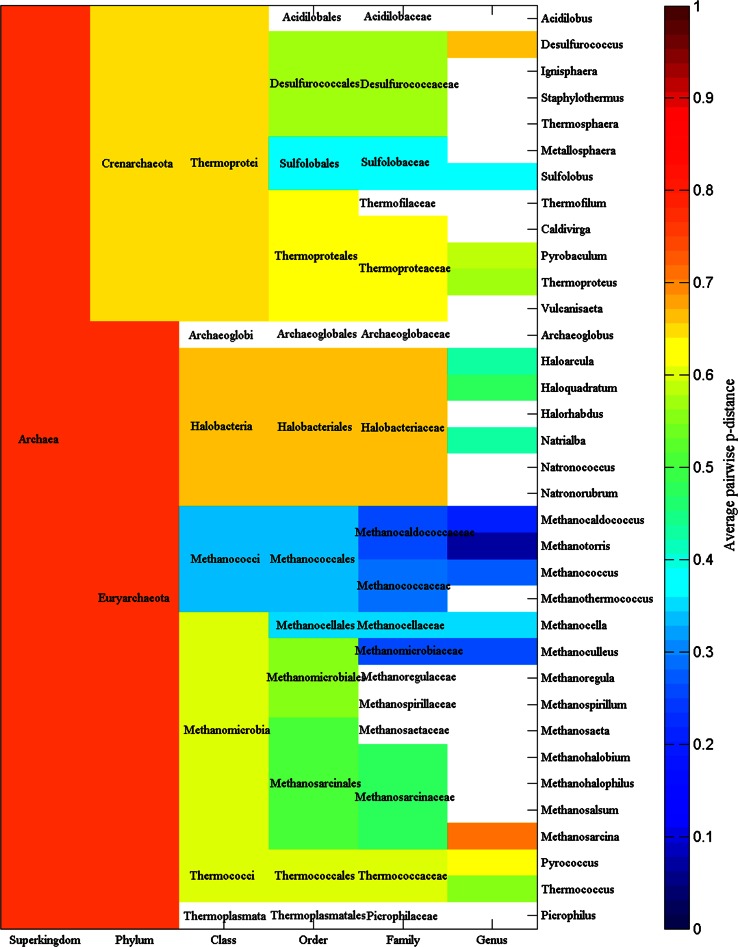
Average *p*-distance of α-amylases for each phylum, class, order, family and genus in archaea. Blank: average *p*-distance is not available

Figure 2 displays the average pairwise *p*-distance of amylases for each phylum, class, order, family and genus in archaea. For example, the average *p*-distance for each of 33 amylases from phylum *Crenarchaeota* is 0.6407, which is the mean value of *p*-distance for any single amylase versus the rest 32 amylases. In fact, the average *p*-distance indicates the divergence of amylases in each taxonomic group, which nevertheless varies greatly across taxonomic groups. For instance, the average *p*-distance is statistically smaller in phylum *Crenarchaeota* than in phylum *Euryarchaeota* (0.6407 ± 0.1228, n = 33 vs. 0.7794 ± 0.0677, n = 55, mean ± SD, *p* < 0.001).

Again, attention can be paid to the average *p*-distance in genus *Thermococcus,* which is an example discussed in Fig. 1. Genus *Thermococcus* can be found in the next to the bottom entry in the right-hand of Fig. 2 and its average *p*-distance is 0.5604, which is smaller than the average *p*-distances of family, order, class and phylum that genus *Thermococcus* belongs to. This provides additional information of why the amylases from genus *Thermococcus* evolved into two clusters in Fig. 1. It is because genus *Pyrococcus*, whose average *p*-distance is 0.6313, from the same family *Thermococcaceae* also evolved into two clusters. Similarly, Fig. 2 gives the clue to find out why some genera have different average *p*-distances from their belonging family, order, class and phylum.

**Fig. 3:**
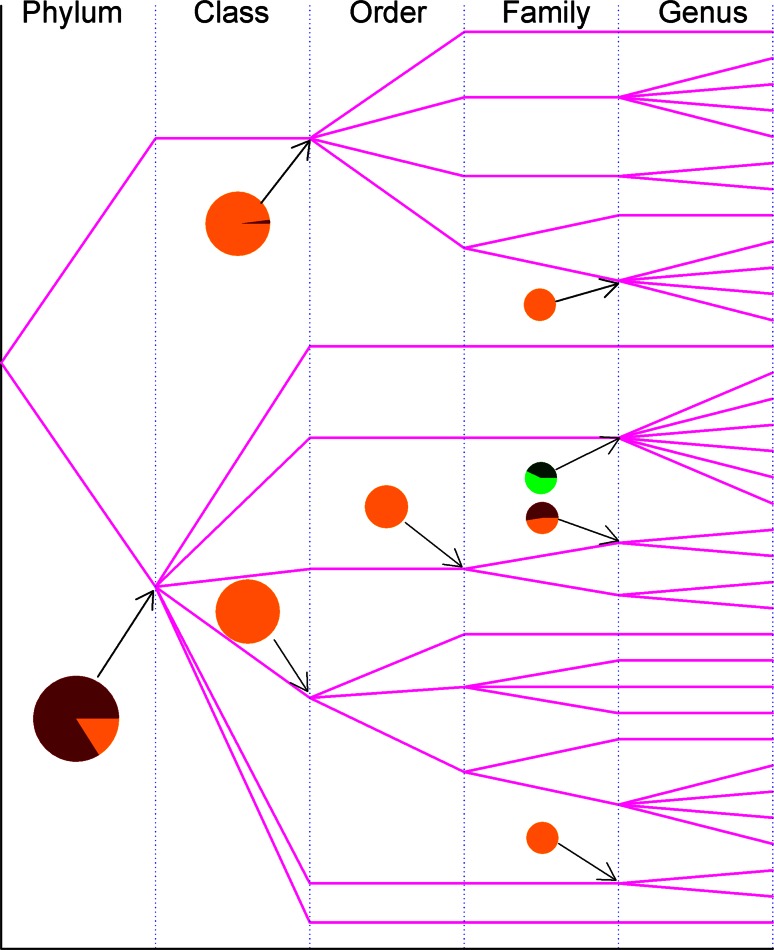
Partitioning of *p*-distance into inter-clan and intra-clan variances for α-amylases from archaea along taxonomic linkage. The bifurcation is the point across taxonomic boundary. *Pies* show the inter-clan variance (*dark* *color*) and intra-clan variance (*bright color*). The *green* and deep *green* pie is an example discussed in “Results section”. Taxonomic names can be found in Supplementary Material Table S2

Figures 1 and 2 already suggest the way to track the flow of genetic materials of amylases, which can transmit both vertically and horizontally. When the variance of *p*-distance is partitioned into inter-clan and intra-clan variances, then new insight can be obtained. Figure 3 illustrates inter-clan and intra-clan variances of archaea amylases, and emphasizes whether the evolution of amylases is likely to be constrained within genus, family, order, and class or across them. This figure can be read as follows: the amylases from archaea (superkingdom) come from two phyla *Crenarchaeota* (upper line) and *Euryarchaeota* (lower line), and the data from phylum *Euryarchaeota* were allowed to compute the inter-class variance (83.98 %) and the intra-class variance (16.02 %), which were marked as dark and bright portions in the pie. The amylases from *Thermoprotei* (upper line at class level) come from four orders, *Acidilobales*, *Desulfurococcales*, *Sulfolobales* and *Thermoproteales*, of which the inter-order and intra-order variances are 2.01 and 97.99 %. The haloadaptation discussed in Fig. 1 is relevant to family *Halobacteriaceae*, and now Fig. 3 delineates its inter-genus and intra-genus variances into 42.78 and 57.22 % in green colored pie. In this circumstance, the haloadaptation in archaea is more likely to occur within family *Halobacteriaceae* because of its large intra-genus variance.

**Fig. 4:**
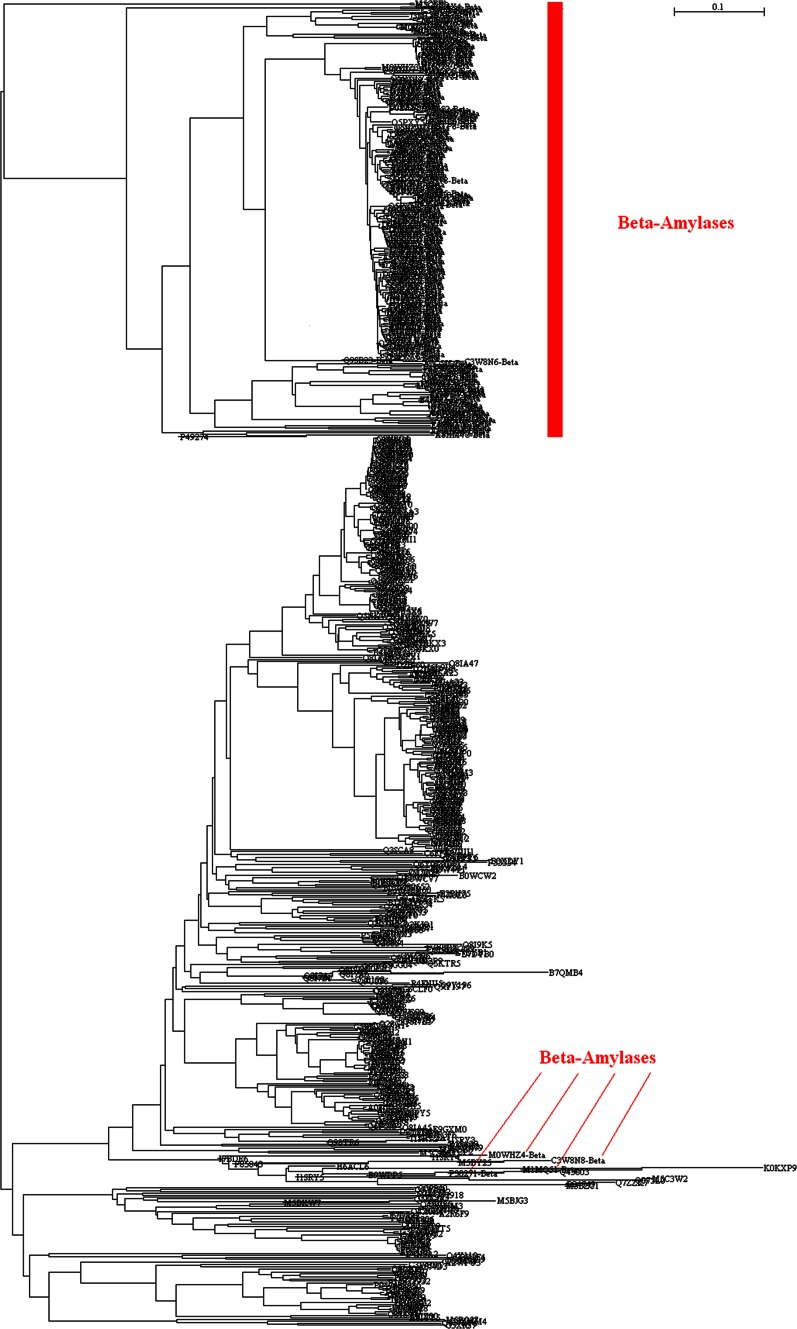
Full phylogenetic tree of 724 amylases from eukaryota. β-amylases distribute in the *upper* cluster marked in *red bar*, and only 4 β-amylases scatter among α-amylases in the *bottom* indicated by *red line*. The full phylogenetic tree in Newick format with maximum likelihood bootstrap values on all branches is available in supplementary materials

Figure 4 demonstrates the phylogenetic tree of 724 amylases from eukaryota. Since the portion of β-amylases in eukaryota (485 α-amylases vs. 239 β-amylases) is far higher than that in bacteria (2545 α-amylases vs. 41 β-amylases), it is interesting and important to figure out how β-amylases co-evolved with α-amylases. Figure 4 provides the clear evidence that β-amylases evolved separately from α-amylases because 235 of 239 β-amylases were exclusively clustered together without any entanglement of α-amylases although two α-amylases, M5CEF1 and M5C2U3, stand at the very top of phylogenetic tree. Similarly distinct phylogenetic branch was also found in *Aspergillus affinis* in the past (Davolos and Pietrangeli 2014). The only possible co-evolution can be noticed for four β-amylases, M0WHZ4, C3W8N8, M1MQ51 and P30271, mixed with α-amylases in the lower part of phylogenetic tree.

**Fig. 5:**
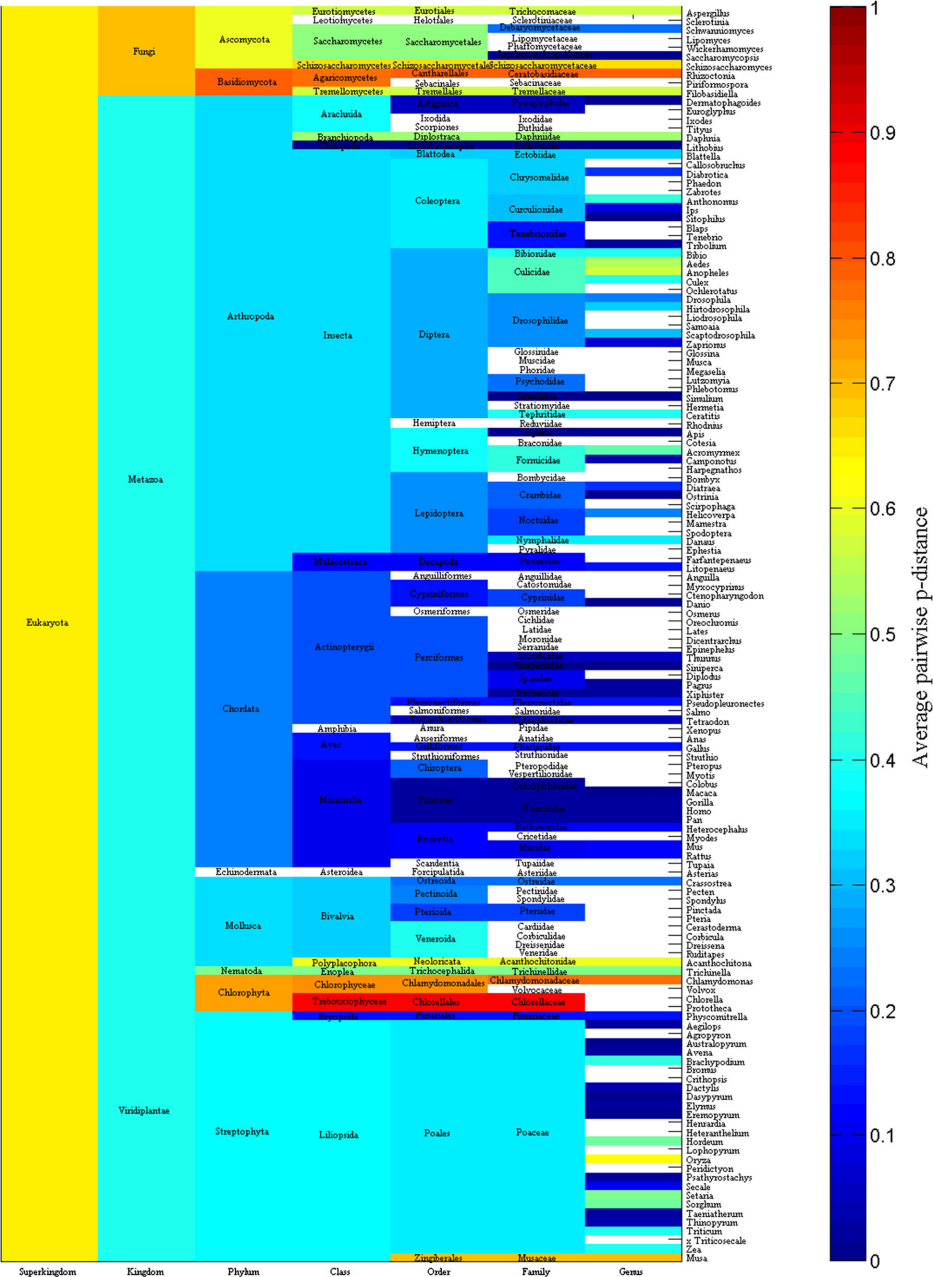
Average *p*-distance of amylases for each kingdom, phylum, class, order, family and genus in eukaryota. Blank: average *p*-distance is not available

Figure 5 pictures the average *p*-distance of amylases for each kingdom, phylum, class, order, family and genus in eukaryota. As a matter of fact, it is only kingdom *Viridiplantae* that has β-amylases, i.e., all 239 β-amylases come from the 274 amyalses in kingdom *Viridiplantae*. Comparisons among *Viridiplantae*, *Fungi* and *Metazoa* result in no statistical difference between *Viridiplantae* and *Metazoa* (0.3929 ± 0.2107, n = 274 versus 0.3924 ± 0.0693, n = 397, mean ± SD, *p* = 0.9632) but statistical difference between *Viridiplantae* and *Fungi* (0.3929 ± 0.2107, n = 274 versus 0.7017 ± 0.1251, n = 53, mean ± SD, *p* < 0.001). Given these results, the co-evolution of β-amylases with α-amylases did not significantly influence the diversity of amylases in kingdom *Viridiplantae*.

**Fig. 6:**
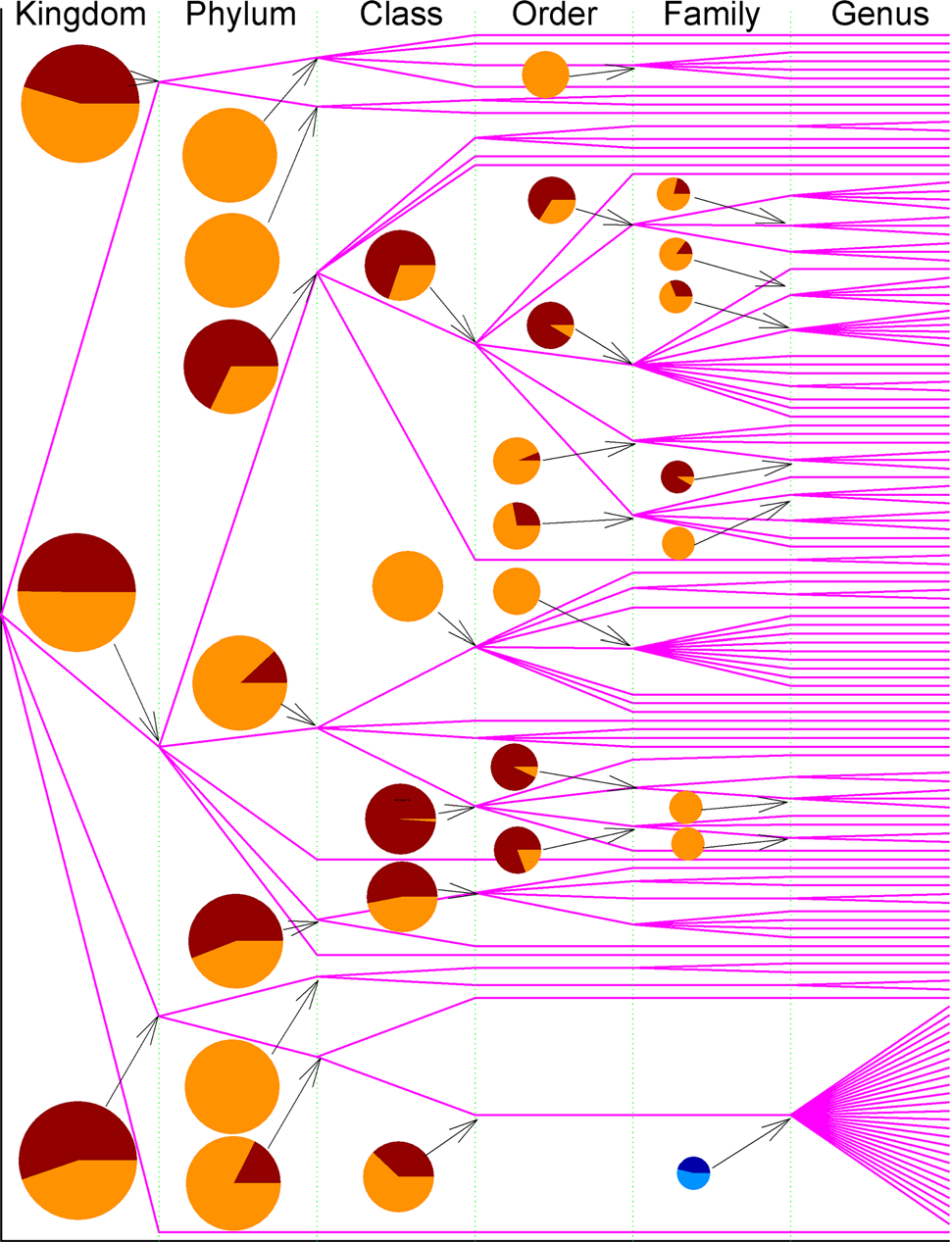
Partitioning of *p*-distance into inter-clan and intra-clan variances for α-amylases from eukaryota along taxonomic linkage. The bifurcation is the point across taxonomic boundary. *Pies* show the inter-clan variance (*dark color*) and intra-clan variance (*bright color*). The *blue* and deep *blue* *pie* is an example discussed in “Results section”. Taxonomic names can be found in Supplementary Material Table S2

Figure 6 exhibits the partitioning of variance of *p*-distance into inter-clan and intra-clan variances in eukaryota. The four β-amylases, M0WHZ4, C3W8N8, M1MQ51 and P30271, which were mixed with α-amylases in the lower part of phylogenetic tree in Fig. 4, belong to four different genera *Hordeum*, *Hordeum*, *Triticum* and *Secale*, but the same family *Poaceae*, whose inter-genus and intra-genus variances are 46.23 and 53.77 % marked in blue colored pie at the bottom of Fig. 6. Thus, the evolution of these four β-amylases could partially be explained as horizontal gene transfer together with vertical gene transfer.

**Fig. 7:**
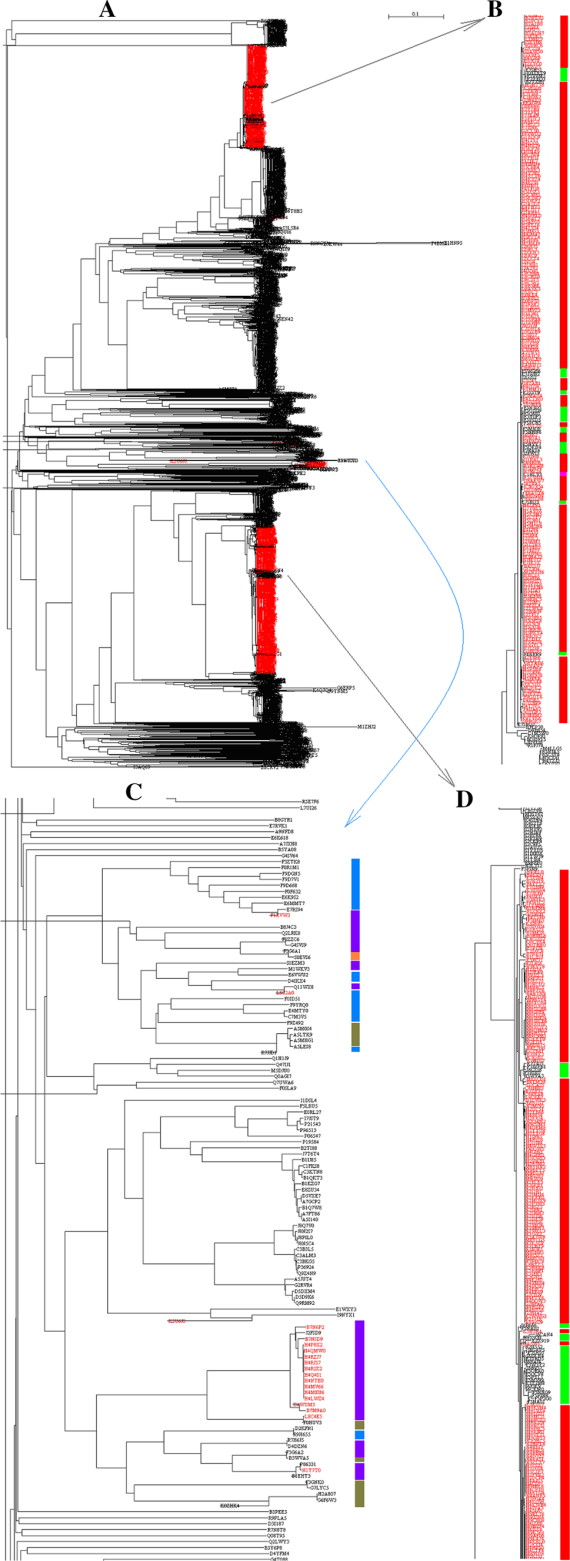
Phylogenetic tree of amylases from bacteria. *Panel a* is the full phylogenetic tree of 2586 amylases from bacteria, where the amylases marked in *red* belong to *E. coli*. *Panel b* is a detailed portion of phylogenetic tree of amylases from *E. coli* (*red bars*) and from genus *Shigella* (*green bars*). *Panel* *c* is a detailed portion of phylogenetic tree of amylases from phyla *Armatimonadetes*, *Bacteroidetes*, *Firmicutes* and *Proteobacteria* were marked in *orange*, *black*, *dark green* and *pink bars*, respectively. *Panel* *d* is a detailed portion of phylogenetic tree of amylases from *E. coli* (*red bars*) and from genus *Shigella* (*green bars*). The full phylogenetic tree in Newick format with maximum likelihood bootstrap values on all branches is available in supplementary materials

Figure 7 highlights the phylogenetic tree of 2586 amylases from bacteria. As seen in Panel A of Fig. 7, the amylases from *E. coli* (marked in red) were formed into two major clusters. It is difficult to determine how many generations of *E. coli* have evolved prior to these two clusters because man-made manipulation could occur at any step of bacterial evolution. However, the incorporated amylase gene into *E. coli* could serve as an indicator to follow the gene flow along the phylogenetic tree in Fig. 7. In Panel B of Fig. 7, a single amylase came from species *Escherichia* sp 3_2_53FAA, C1HLV3, marked in pink color in lower middle position of vertical bar. Meanwhile, there were 23 amylases from genus *Shigella*, E7SH51, K0XRX9, F3VVM2, E7TEQ3, B2TXH1, F3WGB6, E7JWB2, J2G2S2, E2XIT9, F5N3D5, F5QZG9, F5Q6I0, E3Y0F3, F5PSD3, F5PCB5, F5MNJ8, F5NHN6, F5NVY7, F5QKU4, F7RA14, J2FWI3, E7SRQ3 and F4NER9, marked in green color along vertical bar. These 24 amylases entangled with the amylases from *E. coli* represent an example of horizontal gene transfer because their phylogenetic branches came from a single source at the bottom of Panel B. By contrast, an alternative situation can be noted in comparison of Panel C with Panel B, it turns out few amylases from *E. coli*. In Panel D of Fig. 7, thirty-one amylases belong to genus *Shigella*, including E7T4J5, F3WPB1, I6CZI8, F3VDI0, B3WVA3, I6B666, F5NA02, I6CAN4, E2X920, E2X919, B2U552, I6GAP5, E7SHS0, I6DLH4, E7T6D5, I6BAI4, F3W3Y2, I6B957, F5QRA0, F5QCV8, F5PYL1, F5MV99, E3Y7M8, I6FJK9, I6CKM0, F5NNG9, F5PIN8, F5P2G0, F5NA01, I6B5V3, F5P2G1, which were marked in green bars. The explanation for Panel B can be applied to these 31 amylases, e.g. the horizontal gene transfer could occur to get some genetic materials from *E. coli*, and afterwards they were clustered with *E.coli* together.

**Fig. 8:**
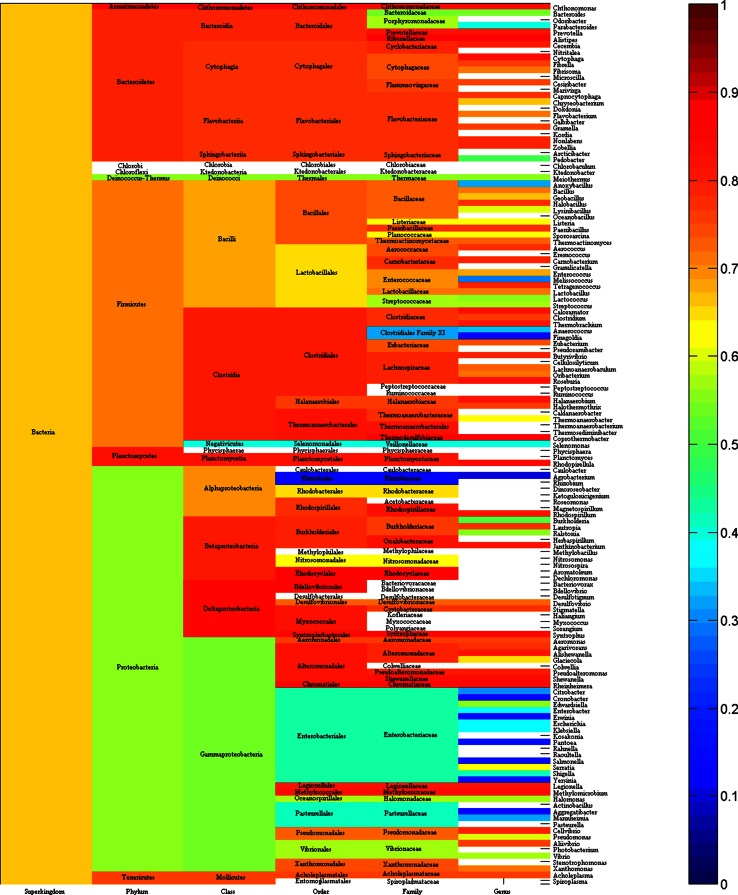
Average *p*-distance of amylases for each phylum, class, order, family and genus in bacteria. Blank: average *p*-distance is not available

Figure 8 depicts the average pairwise *p*-distance of amylases for each phylum, class, order, family and genus in bacteria. It was observed that just a small portion of bacterial diversity can be cultured in laboratory condition (Vester et al. 2014). One might expect to see a small average pairwise *p*-distance under human manipulations. This expectation is true because *E. coli* belonged to genus *Escherichia*, family *Enterobacteriaceae*, order *Enterobacteriales*, whose average *p*-distance is smaller than that in other genera, families and orders as seen in light green color and blue color in the lower part of Fig. 8.

**Fig. 9:**
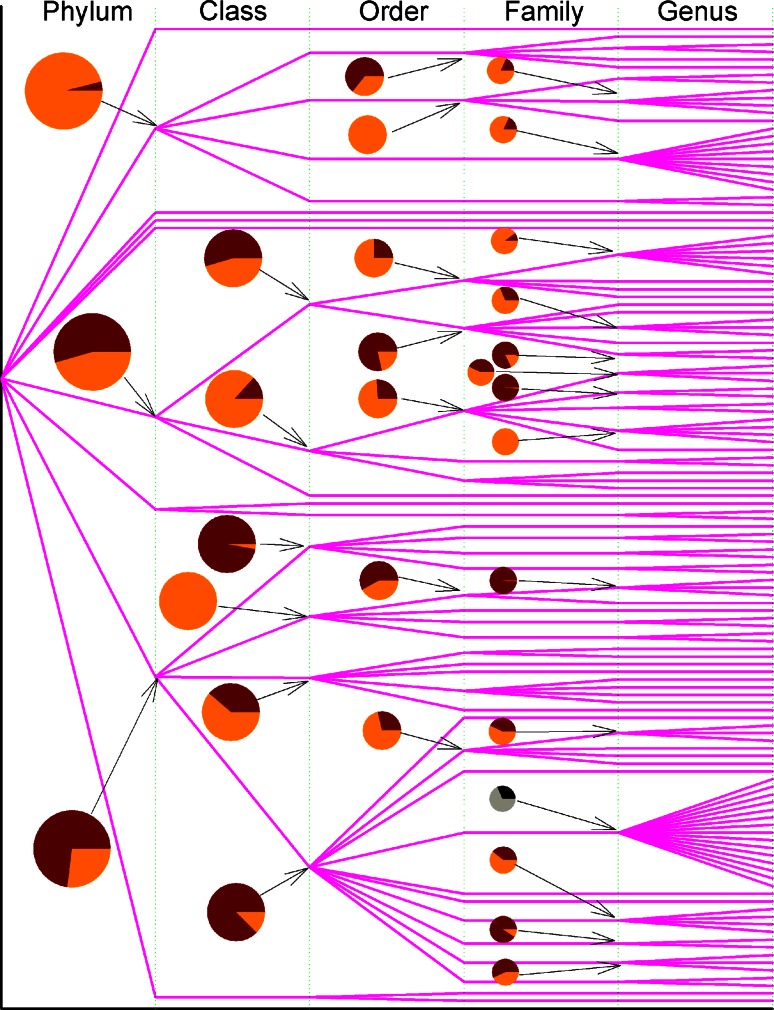
Partitioning of *p*-distance into inter-clan and intra-clan variances for α-amylases from bacteria along taxonomic linkage. The bifurcation is the point across taxonomic boundary. Pies show the inter-clan variance (*dark color*) and intra-clan variance (*bright color*). The *gray* and *black* pie is an example discussed in “Results section”. Taxonomic names can be found in Supplementary Material Table S

Figure 9 outlines the partitioning of variance of *p*-distance into inter-clan and intra-clan variances in bacteria. A follow-up question on engineered *E. coli* is to analyze its inter-clan and intra-clan variances in order to reveal whether man-made manipulation keeps *E. coli* functioning. As seen the gray pie in Fig. 9, the inter-genus variance in family *Enterobacteriaceae* is 31.60 %, to which *E. coli* belongs. This inter-genus variance is indeed not large, so man-made manipulation in *E. coli* has a large probability to survive from generation to generation in biotechnological industries.

**Fig. 10:**
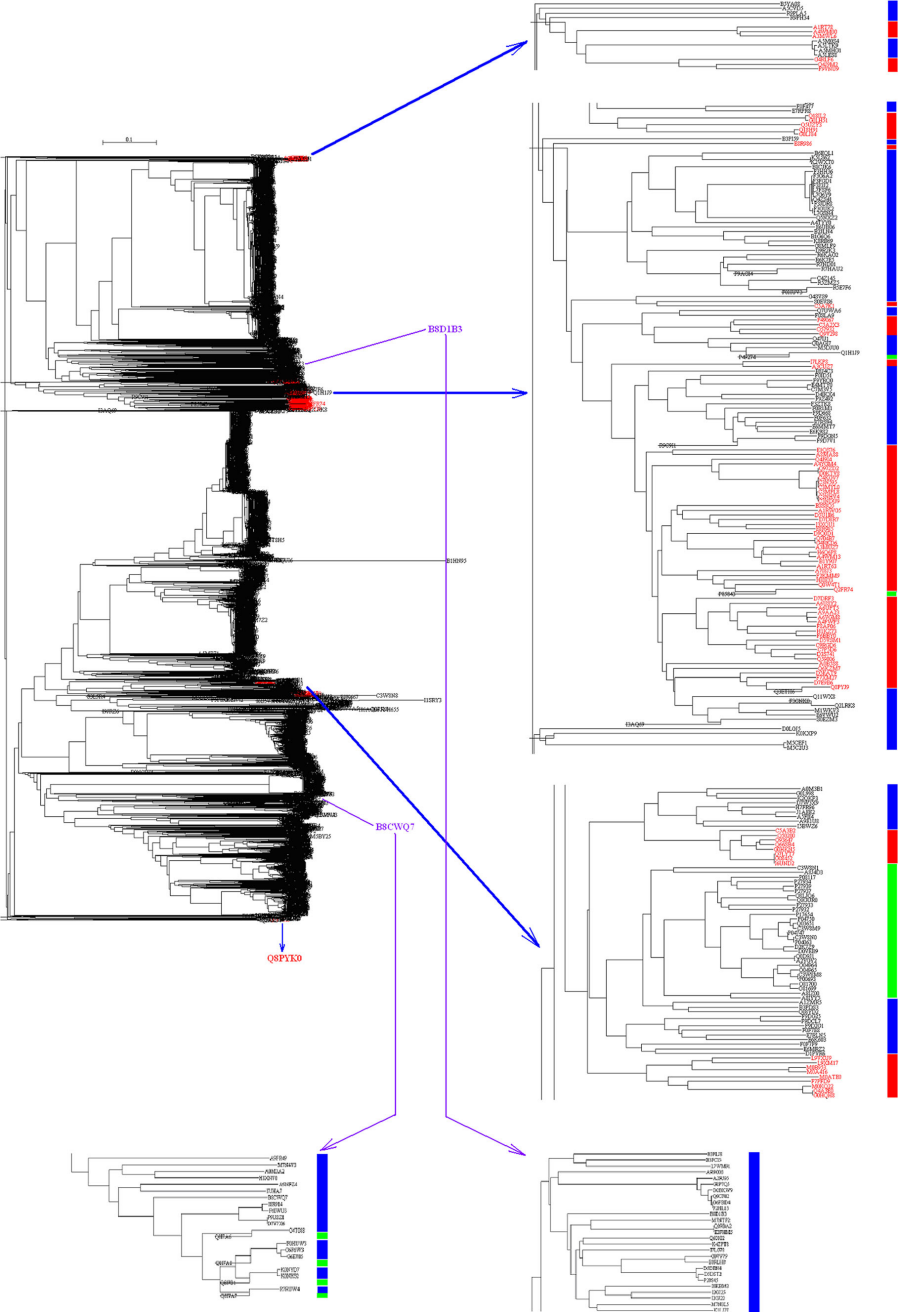
Overall evolution of amylases from archaea, bacteria and eukaryota. Full phylogenetic tree of 3398 amylases was shown in *left panel*. The amylases from archaea were marked with *red*, of which part phylogenetic tree**s** showed the detail distribution in the *right panels*. The detail distribution of two amylases from *Halothermothrix orenii* was shown in *bottom*. The amylases from archaea, bacteria and eukaryota were marked with *red*, *black* and *green bars*, respectively. The full phylogenetic tree in Newick format with maximum likelihood bootstrap values on all branches is available in supplementary materials

Figure 10 describes the full phylogenetic tree of 3398 amylases (left panel). Basically, many interesting evolutionary traces can be tracked in Fig. 10, however emphasis is given to archaea that were marked in red for the sake of space. As seen in the right panel in Fig. 10, the evolution of amylases may undergo vertical and horizontal gene transfer, multiple times and multiple clans, which is evident in light of the coexisting of amylases from archaea, bacteria and eukaryota. Also, glycoside hydrolase family 57 (GH57) contains α-amylases mainly from archaea and bacteria (Janeček and Blesák 2011), in turn this suggests the close relationship of α-amylases between archaea and bacteria. These issues are equally important for bacteria. For example, *Halothermothrix orenii*, which belongs to genus *Halothermothrix*, family *Halanaerobiaceaes*, order *Halanaerobiales*, class *Clostridia,* phylum *Firmicutes*, is an anaerobe for gluconeogenesis and fermentation of ethanol and acetate, whose thermohalophiles are crusial for the conversion of starch into bioethanol (Bhattacharya and Pletschke 2014). There are two amylases from *H. orenii*, M5E0T3 and B8CWQ7, whose locations are far away in the full phylogenetic tree of 3398 amylases as colored in purple. Although these two amylases belong to the same genus, they clearly evolved in a manner of multiple times as demonstrated by purple arrows at the bottom of Fig. 10.

## Discussion

In this study, both phylogenetic and statistical approaches are applied to analyze the evolution of amylases, which are characterized by vertical and horizontal gene transfer, and multi-time and multi-clan evolution.

Different average *p*-distances give a whole picture on the evolutionary history of amylases, i.e., the larger is the average *p*-distance in a taxonomic group, the larger is the probability that a species acquires or loses genetic materials from other group, whereas the smaller is the average *p*-distance in a taxonomic group, the larger is the probability that a species acquires its genetic materials vertically. Nevertheless, different average *p*-distances can be affected by various factors, notably random factors, such as selection of samples. In addition, a small average *p*-distance suggests that a later expansion of amylases in a certain taxonomic group. For example, the average *p*-distance in class *Methanococci* (blue color block in column class in Fig. 2) is 0.3303, which is smaller than those in other taxonomic classes.

Obviously, the larger the inter-clan variance is, the larger the probability is that the evolution would likely cross the taxonomic boundary line. Although the available data are not sufficient to compute inter-clan and intra-clan variances for all taxonomic groups, what was computed indicates that inter-clan variance in archaea is generally not large because dark portions do not account for a major portion in each pie. Really, inter-clan variance can be referenced to the horizontal gene transfer because it represents the chance to pass the genetic materials across taxonomic boundary line.

It ends up with the co-evolution of β-amylases and α-amylases in kingdom *Viridiplantae*, for the reason that their average *p*-distance did not change too much within kingdom *Viridiplantae*. Oppositely, the average *p*-distance in phylum *Chlorophyta* changed significantly (brown color in Fig. 5), whose ratio of α-amylases versus β-amylases is 4/5, and definitely this increase in average *p*-distance cannot be attributed to either α-amylases or β-amylases. In the past, co-evolution was observed in fungus-gardening insects because their lineages shared fungal symbionts with microbes, and co-evolution thus appeared diffuse co-evolutionary relationships, while microbes exhibit none of symbiont sharing, resulting in host-fungus fidelity (Kooij et al. 2014; Seal et al. 2014).

In the analysis on eukaryota amylases, the hypothesis that takes into consideration is an ontogenetic dietary shift from carnivory to herbivory or omnivory, for which family *Stichaeidae* was defined (German et al. 2014). Two species from family *Stichaeidae*, A0FCQ3 and A0FCQ5, were neighbored with D3TJK0, Q5EMQ2, Q8QGJ0, G5EMQ0 and G5EMQ1 from the same order *Perciformes*, and Q9I9H5 from a neighboring order *Pleuronectiformes*, therefore these evolutionary branches in phylogenetic tree could together evolve according to this hypothesis. The lineage-specific expansions were partly driven by directional selection, and gains and losses of genes were lineage dependent (Da Lage et al. 2013). In realty, the intestine environment also plays a role in positive selections of fungal α-amylase in ants (Bacci et al. 2013) as witnessed in local diet (Linton et al. 2014), and gene encoding amylases in marine yeasts versus terrestrial yeasts (Chi et al. 2009).

For the evolution of amylases from bacteria, particular attention is paid to the amylases from *E. coli*, because *E. coli* is not capable to use starch as its carbon source (Rosales-Colunga and Martínez-Antonio 2014) and belongs to non-probiotic bacteria such as *Enterobacter aerogenes* (Das et al. 2014), therefore the involvement of *E. coli* in amylase evolution is mainly driven by the fact that amylase gene was cloned and expressed in *E. coli* because *E. coli* is one of the most widely used microorganisms in biotechnological industries (Tao et al. 2008). This manipulation certainly changes the gene pool in bacteria because 24.05 % (622/2586) amylases come from *E. coli* in the database used in this study. On the other hand, *Lactobacillus*, a genus of probiotic bacteria, is also a bacterium that is cloned and expressed for secreting amylase (Kim et al. 2008; Yoon et al. 2008). Over here, the question is how an amylase gene was incorporated into *E. coli*. This could be explained in two ways: (1) These gene transfers were facilitated by human manipulation because the amylases in *E. coli* come from four phyla *Armatimonadetes*, *Bacteroidetes*, *Firmicutes* and *Proteobacteria*. (2) These gene transfers come from the horizontal gene transfer because of tight contact of *E. coli* with other bacteria in particular environments.

In a broad sense, the study on the evolution of amylases is equal to the study on the evolution of conserved regions in amylases. For example, (α/β) 8 barrel is a conserved the region (Hleap et al. 2013). For another example, α-amylase is a calcium-dependent metalloenzyme whose calcium ion binding regions are subject to evolutionary insertions and deletions (Singh and Guruprasad 2014), so several hypotheses were proposed on gene acquisitions and deletions (Minaya et al. 2013). However, the conversed regions in amylase might have been impacted by the effort in development of inhibitory substances on α-amylase. For instance, *Phyllostachys edulis* produces an inhibitory substance (Yang et al. 2014) and SbAI is an inhibitory gene in potato (Zhang et al. 2014). Still, the access to high- and low-starch diets impacts the evolution of amylase as demonstrated by the number of amylase gene copies (Perry et al. 2007; Santos et al. 2012; Freedman et al. 2014).

It would not be expected that phylogenetic tree would branch in good agreement with taxonomic lineage because recent taxonomic classifications are based on 16s rRNA gene sequences (Krishnan et al. 2012; Sahay et al. 2013; Subhash et al. 2014), while phenotype often results from biochemical tests for amylase (Blackburn et al. 2013). For example, maltose is the main product of starch hydrolysis and an indicator of β-amylase activity (Li and Yu 2012). In this study, the analysis based on amylase taxonomic classification is reasonable because amylolytic enzymes in family GH13 are classified into four groups, i.e., heterologous α-amylase, eukaryotic α-amylases, bacterial and fungal α-amylase and GH13 α-glucosidases (Chen et al. 2012). For this reason, pullulanase whose crystal structure looks like a typical α-amylase structure (Zhen et al. 2014) is not included in this study.

Our data are far from complete if we consider how many species could exist in each superkingdom. The availability of detailed taxonomic lineage imposes the limit for any large-scale studies on the evolution of amylases, and a resultant consequence is the unbalance in available data in each superkingdom, phylum, class, order, family and genus. To a certain degree, this unbalance could cause different interpretations for Figs. 2, 3, 5, 6, 8 and 9. At different taxonomic level, divergence is different. For example, the colors at superkingdom level in Figs. 2, 5 and 8 are quite similar, suggesting a similar rate of mutations in consistent with molecular clock. But the colors at genus level in Figs. 2, 5 and 8 are very different, confirming the difference in lifespan between bacteria/archaea and eukaryota. For Figs. 3, 6, and 9, the partition of variance stratifies variance into vertical and horizontal gene transfers, of which the horizontal gene transfer is primary concern. For various reasons, the horizontal gene transfer depends on many factors, while our analysis concentrates on the horizontal gene transfer within a superkingdom with the hope that model II ANOVA could serve as a method to quantify vertical and horizontal gene transfers.

In this study, we explore the general pattern of amylase evolution with the hope to potentially enhance the successful rate of cloning and expression of amylase gene in different species by employing phylogenetic and statistical approaches to a large dataset. The phylogenetic tree, diversity of average pairwise *p*-distance and its partitions would be helpful for the engineering of new amylases.

## Electronic supplementary material

Supplementary material 1 (DOC 416 kb)
