# Supplementary material for: Analysis on evolutionary relationship of amylases from archaea, bacteria and eukaryota
Source: World J Microbiol Biotechnol. 2016 Jan 8;32:24. doi: 10.1007/s11274-015-1979-y (PMC4706583; doi:10.1007/s11274-015-1979-y)
Supplement: Supplementary file 1 — Supplementary material 1 (DOC 416 kb) [file 11274_2015_1979_MOESM1_ESM.doc]

Table S1 3808 amylases used in this study

| D9Q0D1 E8R985 E8R986 I3XQU1 E0SSQ5 D7D8R7 D5U1B6 A4YGM4 C3MPL8 C3MYL0 C3N595 C3NDU9 C3NHV4 C4KGV7 D0KTV8 F9VNU9 Q4J9I4 Q4J9M2 Q97ZD2 A1RWG5 A8MA88 A1RT63 A1RT78 A3MUZ7 A3MWL6 A4WM00 A4WM13 B1Y907 H6Q6P8 G4RKD6 G4RLF6 Q704B7 E1QS76 F2KMM9 G0HQH8 M0KQ22 Q4A3E0 Q5UZY3 G0LH51 G0LJ84 Q18H91 Q18IL2 F7PFD9 M0A416 M0ATE0 M0B953 L9XM17 L9VXU9 Q59006 C7P5D6 C9RGD6 D3S741 D5VSM1 F6BBY0 H1KZ73 A4FWP3 A6UPT5 A6USY2 A6VGM8 A9AA23 D7DRF3 F8AP06 H8I833 Q0W4T1 A3CUZ7 I7LKP8 A7I8J3 Q2FR74 A0B5S8 D7E9B6 D5EAT9 F7XM27 Q8PYJ9 Q8PYK0 Q9V298 P49067 O57932 I6UND2 O08452 Q7LYT7 C5A2X3 C5A3B2 C5A7K1 G0HKN5 O50200 O93647 Q66SB4 Q6KZM7 S0EVS6 S0EZM3 R5AWF1 B3CDI7 C3QQ37 D0T930 D1JSY4 D7IQJ0 E1WT49 E1YWQ1 E4VYM6 F0R1M1 F3ZTK8 Q5LAX9 F9Z492 R6IAA5 R7JJD1 A6LAI5 C7X7F9 R5ARE7 R7EX23 R7H6J5 R7HNR5 R7J2R6 D1PVR6 D1PXU4 D3I187 E6K603 E6K618 E6K629 E6K9S2 E6MMA8 E6MMT7 E6MRN1 E6MRZ2 E7RLM2 E7RLN5 E7RPR8 E7RS94 E7RSP1 F0F477 F0F632 F0F7E8 F0F7F6 F0F7F9 F0F9Q0 F9D2E8 F9D2G1 F9D668 F9D6T3 F9D7V1 F9D9R6 F9DCL7 F9DCQ5 F9DG25 F9DG62 F9DGN5 F9DIS0 D4IKX4 E4MAA7 K1KXT5 K1L0D8 K1LJ77 I5BWZ6 Q11SE9 Q11WX8 I0KBM3 I0KGT7 I2GEP4 I2GJ22 I2GJ25 I2GP48 A1ZMR5 M7MWR3 M7N3L5 M7N4V3 M7NTF2 M7NTQ8 M7NTT5 C7M3V5 E4MQ63 E4MTY0 F0ID51 F0ID56 F9YRQ0 D7VZ09 D7W3M0 D7W3X9 A2TW00 A5FII4 H7FPC6 H7FR96 H7FSN1 H8XNV0 J1AEE2 K2QKF3 A0M3A2 A0M3A3 A0M3B1 A9E1U8 L7W8H0 L7WBN5 G0L998 G0LCY3 R9H655 A6EH69 Q8KED4 D6TEV5 D3PLX4 I1VWH9 I1VWI0 Q9RM92 Q9Z4N9 R0MR20 R9C0U4 R9C9I1 R9CEN9 A0RGQ2 A1YR25 A5JUT4 A6CIL9 A7DWA9 P00692 P08137 P06278 P20845 P86331 P00691 P36924 P06547 P96513 B0AVX8 B0FYX9 B0LVG1 B0PX91 B1F464 B1UW46 B3GQD0 B3IZB1 B3YY08 B3Z5Z5 B3ZIS2 B5UL87 B7H8M3 B7IQL5 B7JH56 B8Y1H0 C0KWE6 C1EMD1 C3ALM3 C3B3L5 C3BKG5 C3CLI3 C3LAD0 C3P3U6 C6KML4 C7EP44 D5D9K6 D5DAE5 D5DEN4 D5DME4 D5DST2 D5DXF6 D5DXM4 D5DZX3 D5KR60 D5LN31 D8H5G7 D8H5Z1 F4E7B6 G2RVR4 G4F096 G4NTS1 G4NTU0 G4P133 G4P139 G4P8H4 G4P8I0 G4PA17 G4PC62 G8IJA7 G8U6T3 G8UDW3 H2AC11 H8XE78 H9B4I9 H9BPX5 I1W750 I2C144 I3P686 I7FWZ1 I8UJV0 J3UIR0 J3ZVE9 J5NI03 J7JJH4 J8N2I7 J8N5C4 J8PIL0 J8Q7V0 J9QPI9 K4LYT6 K6D344 L0CM04 M1PN43 M1QS41 M1UC85 M1X5B6 M2V2C5 M4KRF2 M5PEA7 N1LSH9 N1LXA1 O82953 Q1EM93 Q208A7 Q3ETH6 Q3ETH7 Q59222 Q5WHK9 Q63EK4 Q65MX0 Q6GWE2 Q6HFU3 Q6HM24 Q81GN4 Q81YJ4 P06279 B7UDC2 D6R179 G9JNH8 I3QII4 J9PXA2 L7ZW44 L8A3Z0 M9YQF0 Q5L238 Q66T78 Q9KWY6 I0JKF1 B1HN95 B1I0B2 Q8ERW2 P21543 E0RL27 E0RLH8 F5LBU5 F5LPK3 G0VV79 G7VZS0 I7JST9 I7L4N4 K4ZPT8 F9DPY6 F9DY49 Q60051 D4YDT5 D4YFM4 D4YI28 F2I4N2 A8UA75 F4BME1 E2YVB9 E6LHW4 F0EIB9 F0ELG5 H8LDY6 H8LE16 J0XLN3 Q3Y2W9 Q3Y3B8 F3Y9E9 H5T5P2 G4L776 B3W999 C0WNT4 C0WP48 C2EJK1 D6S3G3 D6S3G7 D6S3G8 D7V7X6 D7VEC9 D8IM33 E1JME3 E7FSU9 F0HUV3 F0HUW3 F6ITY8 F6IWU3 F9USZ1 G6EV05 G6F6W3 G6F6W4 I8R914 K0NHS2 K0NVC2 K0NVM4 K0NYD7 Q1WSN3 A2RJ95 D2BKW9 D2BR19 F2HL13 F2HLT5 G6FBD4 G8P7Q5 I7JHA7 Q9CF02 R0M3A7 R0M5X2 R0MGS9 R0MHD3 R0N3J3 R0N3N2 R0N6B1 R0NRJ3 R0P4T8 R4ZLD9 R4ZN25 R4ZY59 R7MTQ1 S2V630 S2VPM1 S2VV29 S2VX38 S3MK07 A3CMN5 A5LES8 A5LNG3 A5LTK4 A5LTK9 A5M0S4 A5M7Z1 A5M7Z2 A5MHG1 A5MML6 A5MSZ8 A8AX54 B1ICI2 B1S3J0 B2DKF5 B2DPP1 B2DT61 B2DYC4 B2E4R9 B5E5N9 C1C7Y2 C1CEX0 C1CL90 C1CQX4 C2LRH8 C5WEL9 C5WEM0 C5WF83 D2EQD4 D3H805 D4FTQ3 D6ZS04 D9N6H0 D9NCZ0 D9NLG7 D9NRY3 D9NXD9 E0PDH6 E0PF91 E0PJV3 E0PLJ7 E0PRX4 E0Q0G6 E0TMD1 E1H0S1 E1LKF6 E1LSV4 E1M0I0 E3CDE1 E3CE34 E3CHY9 E3CRD8 E4L404 E6J367 E6KKR4 E6KMH4 E6KN83 E7PUK1 E7S561 E7SC31 E8JN29 E8JT59 E8JYI4 E8K468 E8K5F8 E8K667 E8K6L3 E8K8H7 E8KAE1 E8KPX2 E8KTR9 E8Q7Y1 E9DKJ6 E9FHL9 E9FKQ2 F0FDR0 F0FJY7 F0FR74 F0I120 F0I810 F0IMT9 F0IUJ5 F0VT98 F0VXJ5 F2B6H1 F2BKH7 F2BSG6 F2C6Y7 F2CDJ2 F2CLY3 F2QCP2 F2QDG4 F3L8N5 F3SI27 F3UA14 F3UHY0 F3URW3 F3UY44 F3VIY2 F3VP04 F3WAT0 F3X7G3 F3XD89 F3XKF6 F5U201 F5U9G0 F5X0U1 F5X6E2 F8DI48 F8LKM4 F8LRC5 F8XZD7 F9E0U1 F9E9A3 F9HDL9 F9LW16 F9M0E6 F9MD85 F9MHC7 F9NFN8 F9P093 F9P6F0 F9PDC0 F9Q4K1 G0IAK5 G2GU99 G5JT25 G5JYK0 G5K1X2 G5KB78 G5KIC8 G6EN42 G6EN43 G6ERP3 G6ERP5 G6J4Q2 G6JBF2 G6JFS9 G6JM85 G6JVC4 G6K1E8 G6K679 G6KDT2 G6KK98 G6KRD9 G6KXW6 G6L3K8 G6L9S5 G6LGL7 G6LNC8 G6LTL8 G6M2A9 G6M912 G6MF88 G6MLH5 G6MUS7 G6MYV3 G6N591 G6NBP1 G6NI45 G6NPK8 G6NUP7 G6P3E7 G6P979 G6PFM3 G6PK30 G6PTQ8 G6Q050 G6Q6C1 G6QBZ6 G6QI82 G6QQ34 G6QXL0 G6R2K0 G6RA57 G6RGF5 G6RM78 G6RUY2 G6RZA0 G6S6S6 G6SE70 G6SKL3 G6SS47 G6SYF5 G6T4P4 G6TAY6 G6TEP0 G6TMS7 G6TUQ8 G6TZG0 G6U6J1 G6UBV8 G6UJ80 G6UNQ4 G6UX03 G6V3H0 G6VA19 G6VFK2 G6VMD2 G6VTF1 G6VZD3 G6W548 G6WCB9 G6WIV5 G6WPS6 H2A6A5 H2A807 H6P8S2 H6PBV4 H7GN06 H7GTT6 H7GZE9 H7H5T2 H7HC79 H7HJV2 H7HPP0 H7I1L4 H7I888 H7IB95 H7IIB8 H7IQL0 H7IXJ0 H7J3V5 H7JA83 H7JG23 H7JN07 H7JUI0 H7K0R5 H7K7E2 H7KDE4 H7KKC7 H7KQM6 H7KWR3 H7L3Z7 H7L9R7 H7LG05 H7LJF6 H7LTE7 H7M0W0 H7M6J0 H7MCH9 H7MHW9 H7MPI9 H7MW51 H7N2J8 H7N6P4 H7NE08 H7NIS3 H7NP46 H7NWG7 H7P2M9 H7P979 H7PF03 I0N9F1 I0NQZ4 I0NWQ3 I0SCQ7 I0SHH4 I0SJV1 I0SQF3 I0SQS1 I0SVW6 I2NG89 I5BL47 I6TRE8 I7MXJ1 J0UFJ4 J0UPK6 J0V906 J0VF50 J0VJ09 J0WHM5 J0XG72 J0XV57 J0XW14 J0YJ30 J0YYM4 J0ZMJ6 J0ZTR2 J1B4L8 J1CU23 J1ERH9 J1IHD3 J1JIQ4 J1JME3 J1N8F9 J1NLW8 J1NX30 J1PQR1 J1PR90 J1PT36 J1QER8 J1QQK4 J1QS21 J1QTJ2 J1R7I5 J1RTH4 J1S7K5 J1SCJ6 J1SMA2 J1SV77 J1TE30 J1TFF5 J1TTU0 J1U5F7 J1U8R8 J1UDR3 J1V2B6 J1VBR1 J3JNV0 J7TFG7 J8SY87 K0ZHK4 K0ZJ57 K0ZWE1 K1AKE1 K1B2Z6 K4Q770 K4Q7Q4 K4Q8L5 K4QC79 L9LGR8 L9LH47 M2DLE5 M2DRF0 M2DXC1 M2DZA9 M2E950 M2EAY0 M2EB59 M2EG27 M2EPV6 M2EUS6 M2EVV2 M2F238 M2F5C6 M2FL17 M2FUJ1 M2FVV2 M2FWM4 M2FXP1 M2FZD6 M2G2R5 M2G6J3 M2GAM4 M2GF97 M2GUX1 M2H3T6 M2HN27 M2HPT7 M2HTI1 M2HU48 M2I037 M2I7X1 M2I8P9 M2IFN0 M2IGI3 M2IHA3 M2IS36 M2ISP4 M2J0K7 M2JGW6 M2JNL9 M2JPG2 M2JUB2 M2JUC1 M2JVN0 M2JX95 M2K276 M2K324 M2K4S5 M2KPL5 M2L3Y1 M2LAG6 M2LP80 M2LQQ5 M2LS60 M2LSR2 M2M433 M2MF43 M3JHI5 M3JP13 M4HT93 M4YVX1 M4YX20 M7D329 M7D7S4 M7DBF0 M7DGF9 M7DMX7 M7E1K8 M7EBI4 N0CCY3 Q04JW6 Q3DST4 Q48T01 Q8DPC8 Q8DT08 Q97Q49 Q99ZB3 I7K7X2 I7LG78 R7I7A0 A5I140 A7FT86 A7GCP2 P23671 B1IJ85 B1KZG7 B1Q7W8 B1QKT3 B1QSJ8 B1QTJ9 B1QZE2 B2TI88 B2TQX0 B2V4P9 C1FKS8 C3KTN8 C4IFN1 C4IKY1 C4IM96 C5US89 D5VXE7 D9R2K3 E8ZU34 G7LYC5 G7MAF3 J7T6T4 M1MGI1 M1ZHJ2 M8JB34 M8JCB6 R7RTP8 R7RUW4 B6WB71 F0GV41 D6S8B4 D9PTF2 E1KZT8 F9MZH7 R5E7F6 R5ZJ24 R5ZLB3 R5ZMZ5 R6K1B4 R6K687 R6K7E5 R6KAG2 R7HAE3 R7HAU2 R7N8T8 R7NAF6 R7ND01 C4Z0S9 C4Z145 C4Z431 E6MEA1 R6QTJ9 P30269 E0RWP9 E0S202 Q70JA8 D2KFN1 E6LK24 E6LP88 E6LR58 E6LSE5 R7IW65 Q3LB10 E6UB06 M5E0T3 B8CWQ7 B8D1B3 Q6WUB4 P19584 B5Y6P8 B5YA08 C4V4P2 C9LRQ5 I0IAJ9 F0SLA9 Q7UGI4 Q7UWA6 B8GYR1 G6XV52 H0H4U8 M8ANZ7 K5BXY2 A8LTQ9 F9Y841 A4TYY0 Q2RX34 B1G6Q6 B2JLN4 G8MLF9 K8RB69 E7RVK1 E7RYC7 D8MYY3 D8P297 F6G7E4 M4UIF6 L9P9T3 L9PI35 Q1H1J9 Q0AGI7 M5DJU0 Q5NXZ2 Q47IJ1 E1WXY3 Q6MNM3 S0G6W7 B8J4C3 E6VWU2 M1WKV3 E3FHM5 Q08T95 Q08XX6 Q08YD2 Q09BA2 D0LGJ5 L7UI26 A9FR49 Q2LRK8 Q2LSY2 Q2LV73 Q2LWY3 A0KGI8 A0KHK5 A0KHU4 P22630 P41131 R9PGZ0 R9PH34 R9PH38 R9PLA5 R9PLD2 R9PUI1 H3ZFR2 I9NYX1 K6X974 K6XPS4 K6YT49 K6Z0A8 Q47YS4 P29957 F3BJ35 F3BM55 L7WM91 L8D6Y6 Q15RX1 Q84CG0 B8CJK6 B8CRV2 F7RP81 I1DSL4 I1DXH5 R1FJ74 C1M5W0 D2TJT7 D2TNR9 D4B854 D4BII9 J0VJ20 K8QPJ0 M3DV15 C9Y338 K8A9X9 K8AJR7 K8AX38 K8BES6 K8BPZ4 K8C451 K8C571 K8C8W7 K8CHL9 K8CN53 K8CNV9 K8DM81 K8DPX0 C5BCF3 C5BE62 E0T7C4 D2ZDW9 F5RQZ9 F5RZ80 G0E1T5 I4ZHX6 I6S736 J0MLQ3 J7GHJ8 K4YG34 L8BF16 L8BQ89 R9NGW4 B2VES5 D0FRL2 D2T9M0 D4HVZ7 D4IG50 D8MTL6 E5B6J8 L0WVF9 N0EI19 N0EU31 N0EZ03 N0F9U5 N0FKJ4 N0FWH2 N0G9W9 N0GJF4 R9EES8 A7ZN53 A7ZTB8 A8A1C9 A8A629 P25718 P26612 B1EHT3 B1LJD3 B1LQS8 B2NKT5 B2NXV5 B2NZP7 B2PC13 B2PHH0 B2PRC6 B3A1X5 B3ADN7 B3AGE0 B3AV71 B3AWW9 B3B8Q7 B3BCY5 B3BQ03 B3BRG4 B3C3H5 B3H8F1 B3HH10 B3HML6 B3HVM2 B3HZK6 B3ICS7 B3IIP0 B3IR19 B3WKV0 B3WSS7 B3X832 B3XJ40 B6ZRT6 B7A1B8 B7L6Y6 B7L8U0 B7LP38 B7LTI2 B7M372 B7M3J5 B7M9A0 B7MCI4 B7MES8 B7MVZ2 B7N219 B7N6P2 B7NBS2 B7NEM4 B7NP71 B7NRF3 B7NSD9 C1HLV3 C2DJC8 C2DPT4 C6EEA8 C6UH83 D3GXH9 D3H1K3 D5D3U4 D5D4V4 D7X9H6 D7XPA8 D7Y0Y1 D7YKN6 D7Z623 D7Z8P9 D7ZRF6 D7ZWK3 D8AEW7 D8AVK5 D8B3B6 D8BGK7 D8C583 D8CJT5 D8E6N6 D8ERC5 E0J535 E0R0A1 E1HM71 E1I2F8 E1ISM5 E1JAA9 E1PDY3 E1PJ79 E1RXW8 E2K0E3 E2K2X8 E2KCA4 E2KJR7 E2KMX0 E2KWI5 E2WWG3 E2X1B9 E3XPE1 E3XSB4 E4PAG0 E5ZW04 E6AGR4 E6AUY6 E6B1G7 E6BEC0 E7HDS7 E7HLC3 E7HWU9 E7I238 E7I5F1 E7I7D2 E7IK10 E7INX2 E7J0D7 E7JAA8 E7JKE0 E7JSN6 E7TNS2 E7TTQ2 E7U9J8 E7UB99 E7UGS4 E7UPN0 E8H3D0 E8HGP5 E8HW33 E8I9D9 E8J0R3 E8J2M1 E8Y4B2 E9THX1 E9TVK6 E9UDB8 F1XRX4 F1XVW3 F1XXW9 F1Y210 F1Y9Q1 F1ZIK7 F1ZNX2 F3U663 F4SLG8 F4SQW5 F4T0T8 F4T5E0 F4TFQ8 F4TM20 F4TTW0 F4TZX7 F4UA67 F4UEJ1 F4UNJ7 F4UUP7 F4V365 F4VGI3 F4VKM4 F4VWD2 F4W1C2 F5MAU4 F8YH31 F9CJW4 F9QUT6 G0D4A9 G0D600 G1YAU6 G1YFS6 G1YQC9 G1YWF8 G1Z5N3 G1ZB41 G1ZJD4 G1ZQE6 G2A1L0 G2A5W1 G2A5W2 G2AHB1 G2AM86 G2AW50 G2B142 G2BAK0 G2BFA6 G2BQB7 G2BV65 G2C5D2 G2CAS5 G2CIG9 G2CL50 G2CZS9 G2D5G2 G2EY25 H3KP28 H4HX29 H4ICC0 H4IT17 H4JEL0 H4JMU4 H4K937 H4KI50 H4KXZ4 H4LD17 H4LU44 H4LWZ4 H4MAN1 H4MEU6 H4MSH5 H4MV66 H4N824 H4NPI6 H4NTE0 H4P6I0 H4P8X2 H4PKQ5 H4Q0Q8 H4Q4S1 H4QIE8 H4QMW0 H4QZS5 H4R2Z2 H4RGL3 H4RJ57 H4RX02 H4RZJ7 H4SCA6 H4ST75 H4T8A4 H4TPB2 H4U8I9 H4UQV7 H4V268 H4VMP4 H4W2X3 H4WBL0 H4WUM3 H4WXP8 H4X5J7 H4XLX0 H4Y240 H4YME7 H4Z2U0 H4ZF93 H4ZXI4 H5AEG5 H5AVY6 H5BAL2 H5BRK3 H5C6R4 H5CM81 H5D3Z3 H5DKF2 H5E2W7 H5EK16 H5F0G3 H5FFK6 H5FXT4 H5GD78 H5GTL7 H5HER7 H5HW36 H5IAC8 H5IJZ7 H5IQR1 H5J2A7 H5JPZ5 H5JZV5 H5KF88 H5KVH5 H5L7T7 H5LN01 H5M1L8 H5MGR4 H5N0G9 H5NAN2 H5NQQ8 H5P5H4 H5PKY4 H5Q0J6 H5QFZ8 H5QV85 H5RB26 H6M9I4 H8D9U9 I2IAC9 I2RH75 I2RXE4 I2S8F4 I2SPL3 I2T886 I2TFI7 I2TWK8 I2USC1 I2V6M7 I2VIJ4 I2VTL4 I2W4G9 I2X3C9 I2XKH3 I2XYC0 I2YBF5 I2YNW5 I2YXX4 I2ZDM9 I2ZRU1 I3A3V2 I4SF62 I4T3V5 I4T4A5 I4TAB1 I4TD17 I4UBP6 I4UQ91 I4UQX5 I5DDU7 I5DG76 I5DHC9 I5DW95 I5DWG2 I5DZ77 I5ESE4 I5ETC7 I5F2N6 I5F9N0 I5FD03 I5FGG3 I5G380 I5GCL8 I5GJI4 I5GN45 I5GU36 I5GWC7 I5HGA4 I5HSX5 I5HV53 I5I2T9 I5I7M5 I5I9I3 I5IWJ1 I5J5C5 I5JFE0 I5JHW2 I5JWU1 I5K0E3 I5K1U8 I5K7W4 I5KV97 I5KXD0 I5L6P5 I5LDJ1 I5LFE7 I5LJS9 I5M7X2 I5MG42 I5MKV5 I5MTI7 I5MYL1 I5N8T3 I5NC66 I5NDE5 I5NY92 I5P705 I5PIA6 I5PM29 I5PQ96 I5Q2P7 I5Q8M8 I5QG93 I5QPB5 I5QZ28 I5R6Y6 I5RBI0 I5RPI0 I5RWP2 I5SDU8 I5SFS2 I5SRJ0 I5STY5 I5T224 I5T5H1 I5TQW8 I5TV72 I5U7Q2 I5U929 I5UDE9 I5UHR5 I5UZH4 I5VBH0 I5VC97 I5VMG5 I5VNL7 I5W3N6 I5W965 I5WM22 I5WNP4 I5WY87 I5XDQ9 I5XIJ2 I5XSU3 I5XZ58 I5Y577 I5Y6K5 I5YKK8 I5YTA3 I5ZCZ6 I5ZEQ6 I5ZQ59 I5ZT56 I6FUK5 I6GN66 J2XI22 J2XJZ7 J7QME9 J7QSC3 J7QX77 J7RY53 J9ZIQ9 K0AJI4 K0BP50 K2XLJ0 K2Y727 K2YJA4 K2YWE6 K2Z0L5 K2ZBA5 K2ZD22 K2ZS58 K3A5K5 K3A7G3 K3AEQ6 K3AJX7 K3AKY5 K3ASN8 K3AVP6 K3B296 K3BBD0 K3BJ60 K3BUH2 K3BX82 K3CAI9 K3CE09 K3CSB5 K3DF58 K3DMY2 K3DTE6 K3DUU6 K3DZK0 K3EK64 K3EW26 K3EWZ5 K3F6E5 K3FE81 K3FPB3 K3FWI8 K3G2J7 K3G6N2 K3GD92 K3GR29 K3GT21 K3GVI2 K3H9P0 K3HU67 K3I1G7 K3I368 K3IDW4 K3IFR3 K3IRN6 K3JBV0 K3JK71 K3JMB5 K3JMG4 K3JVI7 K3K017 K3K1A3 K3KAP4 K3KCU7 K3KQG6 K3KQQ4 K3LBK0 K3LH09 K3LN43 K3LV97 K3LXC2 K3R9U4 K3MHV2 K3MTK7 K3NAK9 K3NKR9 K3NLK6 K3P0V9 K3PF54 K3PN42 K3PS56 K3PU79 K3Q1T3 K3Q768 K3QJ93 K3QJS1 K3QXI3 K3RT25 K3RUK1 K3RVS3 K3S722 K3S9P1 K3SAM0 K3SVY5 K3TKE0 K3TL06 K3U0E2 K3U3H4 K3U6J8 K3UAJ3 K3UPC9 K3UTW3 K3V7X8 K4WH75 K5EZI3 K5F878 K5F9W7 K5FFN0 K5GLR7 K5GW52 K5HBP1 K5HEK8 K5HIT1 K5HSD8 K5I654 K5IHQ8 K5IHY7 K5JM73 K5JUH4 K5K8V5 L0XBZ1 L0XDN3 L0XLZ5 L0YIX3 L0YTK3 L0Z1X2 L1A3I4 L1A3W8 L1AG01 L1BGW9 L1BKH5 L1BRY9 L1CPX1 L1CX73 L1D9S7 L1DX29 L1EDV9 L1EL41 L1FCZ4 L1FLN3 L1G049 L1GLZ7 L1H0Z7 L1HIS0 L1R7U7 L1RB52 L5VLZ0 L8BXZ3 L8C231 L8C2A0 L8C4E5 L8CF31 L8CHX0 L8CK67 L8CQ84 L8CQU2 L8YKU8 L8YPA4 L8Z8Q9 L8ZY84 L8ZZG3 L9AAP3 L9B1S2 L9BBX9 L9BLU8 L9CGV1 L9CLI4 L9CLN9 L9DPF7 L9DQL6 L9E947 L9EUN1 L9G3F3 L9GGZ4 L9H0Q4 L9H9I3 L9HFL1 L9HQA5 L9I3E1 L9IK85 L9IMZ8 M2NT78 M2NYH6 M2PJC2 M4JM47 M5HA32 M5HII9 M5HKI8 M8JKL0 M8KFZ0 M8KJT7 M8KVN4 M8L9X4 M8LG71 M8MCJ1 M8NCZ2 M8NJZ9 M8NZJ2 M8P8C5 M8PJP0 M8PJQ0 M8QM02 M8QSG9 M8RCG3 M8RM74 M8S5Z1 M8SNB5 M8SPF9 M8SU75 M8TQB4 M8U5C0 M8ULS1 M8UQR6 M8VFN6 M8VIU8 M8W924 M8WCF6 M8X038 M8XCD1 M8XIQ2 M8XQY2 M8YCG5 M8YNN2 M8Z907 M8ZRA4 M9AF06 M9B5Y4 M9BPY5 M9BVF4 M9C0M1 M9CB08 M9DEI8 M9DRM3 M9E1H7 M9EG30 M9EL16 M9F707 M9G287 M9G8S9 M9GU57 M9H5T1 M9HF40 M9HM92 M9INL6 M9JMF2 M9JZL3 M9JZN4 M9K561 M9L474 M9L9M1 N1SF83 N1SVL0 N1T7T0 N1T8E8 N2DCP4 N2DD38 N2DJ44 N2EI42 N2EJQ4 N2EXI4 N2FLE3 N2FVN2 N2GPX7 N2GQI3 N2HJD2 N2HPT8 N2I1B1 N2IMD4 N2JEA9 N2JZS6 N2KI51 N2KKE0 N2L4S6 N2LET0 N2LII6 N2MQX9 N2MS92 N2MUJ2 N2NZ16 N2P3W3 N2P4C7 N2Q264 N2QAN2 N2QHC5 N2R5M2 N2RSZ5 N2S8N1 N2SF75 N2SMB3 N2TB46 N2TTU8 N2U7S6 N2UC51 N2UE58 N2UFS6 N2VN36 N2VN70 N2VRH7 N2WLL3 N2X588 N2XJQ7 N2XTG8 N2YN02 N2YUL5 N2Z056 N2ZT83 N3A6C9 N3A7W8 N3ATK3 N3BQD2 N3BXG4 N3CBW6 N3CDR1 N3DE32 N3DL48 N3DSV4 N3E6Z7 N3EVT8 N3EZ77 N3FYD7 N3GA15 N3GXX1 N3H323 N3HEL7 N3HQE1 N3III2 N3IP42 N3IV78 N3J5V2 N3K3A8 N3K5Z9 N3KWS3 N3L3U1 N3LBT9 N3M225 N3MHF8 N3NCU2 N3NJR3 N3NKS9 N3P6R9 N3PGA4 N3PUC9 N3QLS6 N3QTS2 N3R848 N3RJX0 N3S8G7 N3SHV0 N3SLE9 N3TD55 N3TDJ0 N3U5T1 N3UAT2 N3UVZ4 N3UXQ9 N3VRF8 N3VV76 N3WN42 N3X5B1 N3XCJ3 N3XU99 N3Y3L2 N3Z1P7 N3ZGS4 N3ZLX7 N3ZS22 N4A4S5 N4ANB5 N4B4L8 N4BH64 N4CDS1 N4CP10 N4D322 N4DEM8 N4E2F0 N4E8C2 N4EB19 N4F0Z8 N4F854 N4FTY6 N4GEE0 N4GIB3 N4GQW3 N4HDP3 N4HP50 N4HU56 N4IV52 N4IZP6 N4JA65 N4JSX4 N4KHL0 N4KQ98 N4KWB4 N4LTF4 N4LWV0 N4MHX3 N4N8C5 N4NTH9 N4P7Y8 N4PM10 N4PV06 N4Q802 N4RAS1 N4RBI2 N4RTG4 N4RZ14 N4SHJ1 N4SVA3 N6WCR8 Q0TBN0 Q0TGR7 Q1R523 Q1RAL3 Q8CVK4 Q8FGL8 R9BFP0 S1T176 S1TJ84 S1TW39 S1U0U8 S1YXD5 S2AU22 S2C9F5 S2CKP4 S2E1H9 S2FZR4 S2HJA9 S2HX92 S3L370 S3L5R4 B5XMX4 B5XPW1 C8SZV8 C8T7K2 G0GK90 G8WMK1 J1TSX8 J1UNZ7 J1UZR5 J1VDK2 J1VQM2 J1X7F1 J1YVV6 J1ZGZ5 J2AS78 J2BRC0 J2C4H5 J2CCV9 J2GR29 J2MC35 J2PSA2 J2Q5D7 J2RCB2 J2STW7 J2U7E8 J2UUX5 J6HHJ1 K4RXN9 K4S7T8 K4SC98 K4SDP4 K4SEJ8 K4SNL6 K6JJF6 M2A0L2 M3ST67 M5GKI0 M5Q836 M5SQ07 M7PSW7 M7QHH2 M7QS20 N9T0A3 J1QNA4 E1SGR1 G9ARC3 H8DS79 H8NZR7 M9VWR5 R7RGQ0 R7RNA8 P26613 B3YDI1 B3YIZ2 B4A0A4 B4A5A9 B4SW69 B4SWL2 B4T858 B4T955 B4TZ60 B4TZJ2 B5C3Y4 B5C469 B5CJ70 B5EX75 B5F2S5 B5FLE0 B5FRW9 B5MHA3 B5MR52 B5MX66 B5N3J0 B5NJA3 B5NWF5 B5P8W8 B5PBZ7 B5PMW7 B5PNR3 B5PRP3 B5Q9J2 B5R4Q1 C9X7V2 E7USY1 E7UZU1 E7V6V3 E7VR69 E7VUL9 E7WG78 E7WMC3 E7WUL7 E7XCG8 E7XHV7 E7XZY2 E7YA74 E7YKZ3 E7Z2A9 E7ZBT5 E7ZJM9 E8A4A0 E8A7X4 E8ALB4 E8AWQ5 E8BE01 E8BKW2 E8CEG9 E8CP62 E8D848 E8DJW1 E8DMN1 E8DR31 E8E1R9 E8EAZ7 E8EMC6 E8EYE0 E8FCL2 E8FY51 E8G577 E8GHZ3 E8GV61 E9A0D5 E9A7X7 F8VMH6 G5RMB7 G9T568 G9THK3 G9TZ33 G9UA70 G9UJT8 G9V4J4 G9VG36 G9VX94 H0L4Q2 H0LM18 H0LQD7 H0M0Y1 H0MEC1 H0MZF6 H0NA58 H1RHV4 H5VGW3 H5VJG6 I0AAB0 I0AEQ4 I0LXS2 I0M058 I0M247 I0NKU5 I0P0S7 I9DVA8 I9DWU0 I9F6C8 I9FLF7 I9FME0 I9GRR4 I9HJX1 I9IG48 I9JRZ6 I9K0Q7 I9K8W9 I9LCA6 I9LWA8 I9T4Z6 I9VVK5 I9XAE6 I9YP73 I9ZH64 J0AJ47 J0AXS6 J0BZ63 J0D7B0 J0DR57 J0ENC6 J0G9A2 J1HFD9 J1HUH8 J1IG30 J1J1G4 J1JIA9 J1KAC9 J1LCB3 J1MZ03 J1P7G9 J1QJX6 J1RI86 J1WPI3 J2B339 J2CQF2 J2DND0 J2F8R2 J2FBI3 J2FN47 J2G7H9 J2HD58 J2I5D7 K0Q9K5 K0QHZ9 K4ZJW6 K5ACZ3 K5AMU3 K5AYC1 K8S4X8 K8SHH7 K8SML4 K8TE28 K8TGH1 K8TYF2 K8UEH3 K8UJQ2 K8UVY7 K8VNF5 K8VYS8 L5WRS6 L5WT08 L5XDX7 L5XK52 L5Y1H5 L5YIU1 L5YTI7 L5Z6K2 L5ZB00 L5ZQQ5 L6AB71 L6AR73 L6AVH0 L6BM17 L6BQU1 L6C0L2 L6C3E4 L6CLQ0 L6CYC3 L6DDM4 L6DPK3 L6E511 L6EHR3 L6EL81 L6EYF2 L6FUD8 L6G4S4 L6G845 L6GS60 L6H0L2 L6H6B2 L6HQP4 L6HVZ3 L6I952 L6IVD5 L6J349 L6JGZ5 L6JYN5 L6K908 L6KJ22 L6L6T6 L6LCD8 L6LVW9 L6M613 L6MIP0 L6MXJ8 L6N675 L6NZC6 L6P5M5 L6PDT2 L6PLK0 L6PX94 L6QER9 L6QJI6 L6RED2 L6RT89 L6RVV9 L6SQT4 L6STF6 L6SX10 L6T8H5 L6TP91 L6TTX7 L6UWJ7 L6UXT0 L6VM57 L6VMA7 L6WJ48 L6X678 L6XR97 L6XUV1 L6Y3G4 L6YIC7 L6YXG7 L6ZJE1 L6ZXG4 L7A648 L7AMS8 L7ATM9 L7B3B4 L9PZS9 L9Q1D9 L9R2B9 L9RD41 L9REI1 L9RR43 L9SA10 L9SI92 L9T6R2 L9TLV4 M3KG44 M3LJY2 M3LM74 M3M575 M4LLG5 M9XL55 N0BZY6 Q8Z2C1 Q8ZL87 S0AB53 S0AET5 D4DZN5 D4DZN6 D4E989 I0QUI6 I3AQ69 L0W2D9 L0W9K9 M3BBV7 B2TXH1 B2U552 B3WVA3 B3WVA5 E2X919 E2X920 E2XIT9 E3Y0F3 E3Y7M8 E7JWB2 E7SH51 E7SHS0 E7SRQ2 E7SRQ3 E7T4J5 E7T6D5 E7TEQ3 F3VDI0 F3VVM2 F3W3Y2 F3WGB6 F3WPB1 F4NER9 F5MNJ8 F5MV99 F5N3D5 F5NA01 F5NA02 F5NHN6 F5NNG9 F5NNR1 F5NVY7 F5P2G0 F5P2G1 F5P2G4 F5PCB5 F5PIN8 F5PIN9 F5PSD3 F5PYL1 F5Q6I0 F5QCV8 F5QKU4 F5QRA0 F5QZG9 F7RA14 I6B5V3 I6B666 I6B957 I6BAI4 I6CAN4 I6CKM0 I6CZI8 I6DLH4 I6FJK9 I6GAP5 J2FSD9 J2FWI3 J2G2S2 K0XRX9 R9EZE1 R9FRR7 R9G4C4 A1JN22 A6BWH5 A7FPA3 A9R4G7 A9Z5S8 A9ZZC4 B0GBE2 B0GTY6 B0H722 B0HJY8 B0HTM1 C4GZB5 C4HFZ6 C4I0D0 E7B5Q0 E7B956 E8NWL8 F4MYE6 F4N1E7 G4KCT6 I6HJB6 I6HYM1 I6IB93 I6IMG4 I6ISN6 I6JHR5 I6JIY1 I6KAX1 I6KBW9 I7MRP8 I7MRQ5 I7NJC4 I7PFU6 I7Q9Z1 I7QSA3 I7RFW1 I7RUP6 I7SDM0 I7U2Z3 I7UJ22 I7UQ37 I7VKU9 I7W648 I7WIE9 I7WKY8 I7XMT8 I7XQK5 I7Y3S2 I7YPM2 I7ZRG1 I7ZTN3 I8AT62 I8AW61 I8AX15 I8BN90 I8C8S2 I8CW38 I8EKD8 I8EQR8 I8ERR6 I8GK18 I8GK94 I8GT83 I8GWD4 I8HNC2 I8IMP1 I8IPD1 I8IQA7 I8JMH0 I8JSI6 I8NB28 I8NI99 I8PX67 I8QV51 K1BY32 L0RJX2 L0RKK9 N1K459 Q1CD79 Q663W5 Q7CFP2 R9SEN3 R9SF93 D5T914 D5TDR4 D5TDR6 Q5ZSI9 Q5ZUX1 Q5ZUX3 G4SV64 G4SVS9 G4T088 E8KFZ4 C6AN88 C9R3K9 A7JQ87 M9X8D3 C9PPF0 B3PCI5 B3PDS3 B3PE18 B3PEE5 B3PEM7 B3PI59 B3PK73 B3PK75 B3PLJ8 I3I6B5 I3I6B6 A5CVD5 F2ZZC6 F3FGD1 F3G6A1 F3G6A2 F3GNK0 F3GUK2 F3HH36 F3J3J2 F3JDR8 F3JS20 L7FSF6 L7G6Y9 L7GSN4 Q4ZS41 B5FDL2 B6EGT4 B6EQL1 Q5E5R9 Q5E8Y4 D0Z2Y0 A1EJN8 A1ENJ1 A1F2U1 A1F4W0 A2P7H4 A2PNU2 A2PSJ7 A3EHK6 A3EKV8 A3ENY1 A3GJF6 A3GRH2 A3H090 A5EYX2 A5F0M5 A6A1Z3 A6A5P0 A6A9F7 A6AN95 A6AQ11 A6AT21 A6AZN2 A6B0W2 A6B5S1 A6B600 A6XQG1 A6XRJ9 A7JXF7 A7JXH8 A7K2C2 B8K415 C2C8H0 C2HZT7 C2I8D9 C2IGC5 C2ISR9 C2IT66 C2J223 C2JBA2 C3LUS0 C3LWC4 C3NWD6 C6S1M8 C9NTF6 C9P5E7 C9P882 C9PGU1 C9QAG0 C9QD61 C9QE75 D0GYQ6 D0H787 D0HCU0 D0HNY5 D0HUJ7 D0IHB5 D0M4D7 D0MCN0 E1CUZ7 E1CWX3 E1CX33 E1CYP4 E1DAE6 E1DF36 E1DHI9 E1DQA4 E1DU23 E1DV60 E1ECL2 E8VVY1 E8VW46 F8YS92 F8YTN2 F8Z2T3 F8Z4A9 F8ZEQ7 F8ZG79 F8ZP04 F8ZQE5 F9A4H5 F9A5V3 F9AFV9 F9AGI4 F9AGQ7 F9AL07 F9ANA2 F9AXF2 F9AXV3 F9BFK5 F9BGN4 F9BQM2 F9BS65 F9BSM9 F9BU57 F9CAR4 F9CCR1 F9TNM5 G6YZS6 G6Z0N6 G6ZIT9 G6ZJP9 G6ZMC0 G6ZNA9 G6ZXL4 G6ZYP8 G7ABX7 G7AD05 G7AHJ1 G7AIE8 G7B209 G7B306 G7BCH1 G7BDJ9 G7BJ24 G7BKJ2 G7BPS1 G7BR50 G7C1Q7 G7C2T9 G7TXT7 J1C387 J1C496 J1C6E4 J1D165 J1D4B3 J1DBZ8 J1DDA5 J1E769 J1ECJ8 J1EJI0 J1FN29 J1FU89 J1G113 J1G6U2 J1M3C5 J1NX88 J1PUM8 J1VS23 J1W4A3 J1W4S7 J1WKT3 J1WSF4 J1WTS2 J1XCL3 J1XE01 J1XTQ6 J1Y1F0 J1YG67 J1YHB1 J1ZAA8 J1ZD66 J1ZF74 J1ZNL9 J2A998 K2SXM6 K2TQE1 K2TTZ0 K2TWF3 K2U7Z9 K2UGH4 K2ULL6 K2UNH7 K2V084 K2VA65 K2VH00 K2VJ21 K2VJ79 K2VL15 K2VZ07 K2W268 K2W7G5 K2W900 K2WB30 K2WGT6 K2WXT0 K2WY37 K2X0H6 K2X779 K2XIW2 K2XLW7 K2XQ17 K2XRG5 K2XTX0 K2Y333 K2YFX7 K5LS62 K5RI47 K5RYT8 K5S3G2 K5S8D1 K5SH02 K5SKD2 K5TC02 K5TF37 K5TMG7 K5TS02 K5U1W3 K5U4U3 K5UJA6 K5UKP1 K5V9F5 K5VH06 K5VK97 L8QMH1 L8QNN3 L8QVH3 L8QXK1 L8R6D1 L8R735 L8RIR9 L8RK38 L8RNK2 L8RRN6 L8RTP0 L8SBM5 L8SDZ1 L8SMQ1 L8SP01 L8SVL3 L8SX62 L8T4N3 L8TA87 L8TER6 L8TFR4 M0PW10 M7FR69 M7G8W0 M7GBJ3 M7GD03 M7GDP0 M7GEI5 M7GGD6 M7GJ01 M7GKG4 M7GN56 M7GNH9 M7H480 M7HHQ9 M7HJ61 M7HQS4 M7HSU6 M7HZV4 M7I1R6 M7ICM8 M7ICZ0 M7ID70 M7IDR0 M7IDW7 M7IDX3 M7IGH1 M7IP17 M7IVK0 M7J3H8 M7J4Y0 M7J5R3 M7J942 M7JAJ2 M7JB92 M7JBB7 M7JDS9 M7JK51 M7JKI4 M7JPI3 M7K2H0 M7K5U7 M7K8E3 M7KDF0 M7KK43 M7L8X8 M7LD56 M7LIC6 M7LM40 M7LR54 M7LRN7 M7LSN7 M7M0Q6 M7M455 M7M7U3 M7MEQ3 M7MN65 Q1VBR8 Q8D4Q8 Q8D5L1 B2FUJ5 B0RV82 Q60102 Q847N0 A9NFD8 A9NFZ3 A9NFZ4 A9NG03 Q6XK11 A2QL05 A2QW02 A2QYT9 A2R6F9 P30292 Q02905 P56271 P0C1B3 P0C1B4 Q02906 B0FZ76 G3XMM3 G3XMM4 G3XQD9 G3XRS7 G3XUI2 G3Y2F4 G3YCJ0 I7HCZ7 Q4WI35 Q4WIT5 Q4WPQ9 Q4WRT6 Q4X0H4 Q4X110 Q7LV45 C0LW29 P19269 Q08806 E5KJ07 Q01117 K0KXP9 P21567 E7D897 O74922 Q09840 Q9Y7S9 O42918 O14154 M5BJG3 M5BKW7 M5BQS7 M5BY25 M5BZC1 M5BZF7 M5C0W9 M5C2U3 M5C3W2 M5C9M7 M5CEF1 G4TCP2 Q5K993 Q5KGI6 Q9Y197 P49274 Q9Y196 B7QMB4 P85843 E9GG04 E9GXM0 D2YVN2 Q8I9P9 Q2KJQ1 Q2L7A6 Q5KTR5 Q9U406 Q9GU27 O97396 Q9N2P9 Q8I9K5 Q8I9K6 E7DYB0 E7DYB1 E1B2Q9 E7BLI2 Q8I9Q7 P56634 P09107 I7BDR6 Q26854 Q26855 Q6Y0Z2 Q86N60 P53354 O02652 D4N3A2 Q17023 Q17059 B0W4Y1 B0WCV7 B0WCV8 B0WCW1 B0WCW2 B0WEL4 B0WKW9 B0XDY1 B0XFZ8 Q23767 Q16924 Q9NKZ1 Q9NKZ2 Q9NKZ3 Q9NKZ4 Q9NKZ5 Q9U5X5 Q9U5X6 Q23834 Q23835 O18345 P54215 P08144 P83833 P81641 Q9BN01 O18344 O77011 O77020 O77019 O76284 Q9NJN8 O77021 O77012 Q9NJP0 O76265 Q9GQV3 O77013 O76262 O77014 O18408 O77015 O77022 O18552 O76261 O77016 O76459 O18420 O76260 O77018 Q9NJN7 O76263 O76264 B3MFX1 B3NP13 B4HMI1 B4HMI3 B4MDZ5 B4P8G9 B4QAQ6 B5DZS7 D2IP59 D2IP63 G3E6D5 G3E6D6 G3E6D7 G3E6D8 G3E6D9 G3E6E0 G3E6E1 G3E6E2 G3E6E3 G3E6E4 G3E6E5 G3E6E6 G3E6E7 G3E6E8 G3E6E9 G3E6F0 G3E6F1 G3E6F2 G3E6F3 G3E6F4 G3E6F5 G3E6F6 G3E6F7 G3E6F8 I6L932 I6L933 I6L934 I6L935 I6L936 I6L9L2 I6L9L3 I6L9L4 I6L9L5 I6L9L7 I6L9L8 I6L9L9 I6L9M0 I6L9M1 I6L9M2 I6L9M3 I6L9M4 I6L9M5 I6L9M6 I6L9M7 I6L9M8 I6L9M9 I6L9N0 I6L9N1 I6L9N2 I6L9Q3 I6L9Q5 I6L9Q7 I6L9Q9 I6L9R4 I6L9U3 I6LA22 I6LA23 I6LA24 I6LA25 I6LA26 I6LA27 I6LA28 I6LA29 O44204 O77407 Q23932 Q24609 Q24610 Q24611 Q24613 Q24642 Q24643 Q24644 Q24675 Q24676 Q24737 Q27609 Q27610 Q27611 Q27612 Q27863 Q27891 Q27923 Q2VY98 Q2VY99 Q2VYA0 Q2VYA1 Q2VYA2 Q2VYA3 Q5NKX0 Q5NKX1 Q5NKX2 Q5NKX3 Q5NKX4 Q5NKX5 Q5NKX6 Q5NKX7 Q5NKX8 Q5NKX9 Q5NKY0 Q5NKY1 Q5NKY2 Q5NKY3 Q5ZR46 Q8IA40 Q8MLX9 Q8MLY6 Q8MM40 Q8MM52 Q8MM80 Q8MM99 Q8MX51 Q8MX52 Q8MX59 Q8MX66 Q8MX67 Q8MY47 Q8MY48 Q8MY49 Q8MY50 Q8MY51 Q8MY52 Q8MY53 Q8MY54 Q8MY55 Q8N0P5 Q8N0P6 Q8N0P7 Q8N0P8 Q8N0P9 Q8N0Q0 Q8N0Q1 Q8N0Q2 Q8N0Q3 Q8N0Q4 Q8N0Q5 Q8N0Q6 Q8N0Q7 Q8N0Q8 Q8N0Q9 Q8N0R0 Q9BH28 Q9BH74 Q9BN00 Q9BN02 Q9BN06 Q9BN07 Q9BN08 Q9BN09 Q9GRF6 Q9N651 Q9N6Q7 Q9NKY5 Q9NKY6 Q9NKY7 Q9NKY8 Q9NKY9 Q9NKZ0 I6L9U4 Q5NKW7 Q8IA38 Q5NKW8 A8IF87 D2IP60 Q5NKW9 Q8MY46 D2IP57 D2IP58 D2IP62 Q3SCA8 A4UUI1 Q8I9Q2 Q9XZ45 C6FFT8 D1FPT5 D1FPT6 E5LCR9 Q8IA47 Q8IA48 R4FNU5 Q9U8X5 Q8N0N7 B9W4L8 F4X0E0 F4X0E1 E2AS66 E2AS67 E2BN75 C7EMF0 Q7YXJ3 Q7YXJ4 Q7YXU5 Q25590 Q25592 A9XTK5 B1NLD3 B1NLD4 F6K706 Q8IA46 G6CIW2 G6CLF0 G6CLF1 B8Y698 Q8I7B1 Q9U0F6 Q9U0F7 Q9U0F9 Q26193 Q8I7A6 Q8I7A7 Q8I7A8 Q8QGW2 A5JTT8 D0EM61 Q6P5J0 Q7SYL6 C1BKW3 Q7ZZK7 Q8QGJ0 Q98TR6 D3TJK0 B9WPP5 G5EMQ0 G5EMQ1 D3TJH5 E1U2Z0 B6CGL7 G5EMQ2 Q5XPY5 A0FCQ3 A0FCQ5 Q9I9H5 Q9I9H6 A0SEG1 Q8QFS2 Q8UWE3 Q8UWE4 Q8UWE5 Q28G41 R0L136 Q6JG52 Q98942 P83053 L5K5S8 L5LXA1 A8HDI0 F7GYU4 H9EX43 I2CT82 A8HDG5 A8HDH1 A8HDH7 P04745 P19961 P04746 B7ZMD7 Q53F26 A8HDG3 A8HDG8 A8HDH0 A8HDH3 A8HDH5 G5AZM7 G5BKY2 Q2V6H2 P00687 P00688 P00689 Q5I0L0 Q99N59 L9KVU7 Q8IA45 I1SRY2 K1Q3A1 K1QDY3 K1QM72 K1R3F6 O02622 Q8I080 Q8I0T4 Q8I7E4 Q8I7E5 Q8I7E6 Q8I7E7 Q8I7E8 Q8WSG9 Q8WSH2 P91778 K9L927 F8RNZ9 F8RNZ6 D2YVN8 Q8I9P8 H6ACL6 I1SRY3 I1SRY4 I1SRY5 E5S9H2 E5SA29 A8HMV0 A8HW34 A8IMV2 A8IYY5 A8IZ00 A8J4D3 D8TKA0 E1ZGI6 Q075L0 A9RQY6 A9SR20 A9THN6 A9TTT8 Q5PXX5 Q5PXX6 Q5PXY0 Q5PXY1 Q5PXY2 Q5PXY3 Q5PXY4 Q5PXY5 Q5PXX4 Q5PXX8 Q5PXX9 O81699 O81700 I1GTP8 I1GTP9 I1GTQ0 I1H9W3 Q5PXT7 Q5PXX7 D0VEB9 D2K7Z9 Q5PXX2 Q5PXX3 A9UGL2 A9UGL3 A9UGL4 A9UGL5 A9UGL6 A9UGL7 A9UGL8 A9UGL9 A9UGM0 A9UGM1 A9UGM2 A9UGM3 A9UGM4 A9UGM5 A9UGM7 A9UGN8 A9UGN9 A9UGP0 A9UGP1 A9UGP2 D0EKB5 D0EKB6 D0EKB7 D0EKB8 D0EKB9 D0EKC0 D0EKC1 D0EKC2 D0EKC3 D0EKC4 D0EKC5 D0EKC6 D0EKC8 D0EKC9 D0EKD0 D0EKD1 D0EKD2 D0EKD3 D0EKD4 D0EKD5 D0EKD6 D0EKD7 D0EKD8 D0EKD9 D0EKE0 D0EKE1 D0EKE2 D0EKE3 D7R4Q7 D7R4Q8 D7R4Q9 D7R4R0 D7R4R1 D7R4R2 D7R4R3 D7R4R5 D7R4R6 D7R4R7 D7R4R8 D7R4R9 D7R4S0 D7R4S1 D7R4S2 D7R4S3 D7R4S4 D7R4S6 D7R4S7 D7R4S8 D7R4S9 D7R4T1 D7R4T2 D7R4T3 D7R4T4 D7R4T5 D7R4T6 D7R4T7 D7R4T8 Q5PXV0 Q5PXV1 Q5PXV2 Q5PXV3 Q5PXV4 Q5PXW9 Q5PXX0 Q5PXX1 Q5PXW8 Q5PXW7 Q9SB23 Q9SBH7 A8CFR3 A9UGM9 A9UGN0 A9UGN1 A9UGN2 A9UGN3 A9UGN4 A9UGN6 A9UGN7 P00693 P04063 P04747 P04748 P04749 P04750 P82993 P16098 C1IIM6 C3W8M8 C3W8M9 C3W8N0 C3W8N1 C3W8N4 C3W8N5 C3W8N6 C3W8N7 C3W8N8 D0EKB2 D6BU16 D6BU17 E0W6Z7 F2DKW4 F2DM00 F2DY58 F2EFV1 M0UST1 M0UST2 M0UST3 M0W7M7 M0W916 M0W917 M0W918 M0WHZ3 M0WHZ4 M0WHZ5 M0WHZ6 O04964 O04965 O23978 Q03651 Q4VM10 Q4VM11 Q5PXV8 Q5PXV9 Q5PXW0 Q5PXW1 Q5PXW2 Q5PXW3 Q5PXW4 Q5PXW5 Q5PXW6 Q6SNP7 Q84T19 Q84T20 Q9AVJ8 Q9FSI3 Q9FUK6 Q9FUK7 Q5PXU0 A2XCC5 A2YMB7 A2Z8A8 A3ADZ2 A3C5J5 A3C5J7 P17654 A2YGY2 Q0D9J1 P27932 P27937 P27939 P27933 P27934 A2WPU3 Q0JMV4 B8B797 C7J4G6 I1P7F5 I1QBC2 I1QBC3 I1QV87 I3QD77 J3LJV4 J3MLS0 J3MLS2 J3N387 Q10RZ1 Q42989 Q42990 Q43003 Q6Z5B2 Q6Z5B7 Q9AV88 Q5PXV7 Q5PXV5 Q5PXV6 P30271 Q08335 Q5PXU6 Q5PXU7 Q5PXU8 Q5PXU9 K3ZRZ8 K4A2Q0 K4A7U0 K4A838 C5WYV3 C5WZD6 C5XAT3 D3JBK2 Q5PXU2 Q5PXU3 Q5PXU4 Q5PXU5 D0EKB3 Q5PXU1 Q9ZR48 P08117 P93594 D0EKB4 M1MQ51 Q5PXT8 Q5PXT9 I7C0A2 Q9SYS1 P55005 B4FJF7 B4FW64 B6SXN4 B6SYP0 C0P5G0 D4PB89 M0RGQ8 M0S7U7 M0SX91 M0UD97 Q3S4X4 Q8GUR0 Q8LJQ6 |
| --- |

Table S2 Taxonomic classification of 92 amylases from archaea used in this study

| Phylum |  | Class |  | Order |  | Family |  | Genus |  |
| --- | --- | --- | --- | --- | --- | --- | --- | --- | --- |
| Crenarchaeota | 33 | Thermoprotei | 33 | Acidilobales | 1 | Acidilobaceae | 1 | Acidilobus | 1 |
| Euryarchaeota | 55 | Archaeoglobi | 1 | Desulfurococcales | 6 | Desulfurococcaceae | 6 | Desulfurococcus | 3 |
|  |  | Halobacteria | 14 | Sulfolobales | 12 | Sulfolobaceae | 12 | Ignisphaera | 1 |
|  |  | Methanococci | 14 | Thermoproteales | 14 | Thermofilaceae | 1 | Staphylothermus | 1 |
|  |  | Methanomicrobia | 12 | Archaeoglobales | 1 | Thermoproteaceae | 13 | Thermosphaera | 2 |
|  |  | Thermococci | 13 | Halobacteriales | 14 | Archaeoglobaceae | 1 | Metallosphaera | 1 |
|  |  | Thermoplasmata | 1 | Methanococcales | 14 | Halobacteriaceae | 14 | Sulfolobus | 11 |
|  |  |  |  | Methanocellales | 2 | Methanocaldococcaceae | 7 | Thermofilum | 1 |
|  |  |  |  | Methanomicrobiales | 4 | Methanococcaceae | 7 | Caldivirga | 1 |
|  |  |  |  | Methanosarcinales | 6 | Methanocellaceae | 2 | Pyrobaculum | 8 |
|  |  |  |  | Thermococcales | 13 | Methanomicrobiaceae | 2 | Thermoproteus | 3 |
|  |  |  |  | Thermoplasmatales | 1 | Methanoregulaceae | 1 | Vulcanisaeta | 1 |
|  |  |  |  |  |  | Methanospirillaceae | 1 | Archaeoglobus | 1 |
|  |  |  |  |  |  | Methanosaetaceae | 1 | Haloarcula | 4 |
|  |  |  |  |  |  | Methanosarcinaceae | 5 | Haloquadratum | 4 |
|  |  |  |  |  |  | Thermococcaceae | 13 | Halorhabdus | 1 |
|  |  |  |  |  |  | Picrophilaceae | 1 | Natrialba | 3 |
|  |  |  |  |  |  |  |  | Natronococcus | 1 |
|  |  |  |  |  |  |  |  | Natronorubrum | 1 |
|  |  |  |  |  |  |  |  | Methanocaldococcus | 5 |
|  |  |  |  |  |  |  |  | Methanotorris | 2 |
|  |  |  |  |  |  |  |  | Methanococcus | 6 |
|  |  |  |  |  |  |  |  | Methanothermococcus | 1 |
|  |  |  |  |  |  |  |  | Methanocella | 2 |
|  |  |  |  |  |  |  |  | Methanoculleus | 2 |
|  |  |  |  |  |  |  |  | Methanoregula | 1 |
|  |  |  |  |  |  |  |  | Methanospirillum | 1 |
|  |  |  |  |  |  |  |  | Methanosaeta | 1 |
|  |  |  |  |  |  |  |  | Methanohalobium | 1 |
|  |  |  |  |  |  |  |  | Methanohalophilus | 1 |
|  |  |  |  |  |  |  |  | Methanosalsum | 1 |
|  |  |  |  |  |  |  |  | Methanosarcina | 2 |
|  |  |  |  |  |  |  |  | Pyrococcus | 6 |
|  |  |  |  |  |  |  |  | Thermococcus | 7 |
|  |  |  |  |  |  |  |  | Picrophilus | 1 |

Table S3 Taxonomic classification of 724 amylases from eukaryota used in this study

| Kingdom |  | Phylum |  | Class |  | Order |  | Family |  | Genus |  |
| --- | --- | --- | --- | --- | --- | --- | --- | --- | --- | --- | --- |
| Fungi | 53 | Ascomycota | 39 | Eurotiomycetes | 26 | Eurotiales | 26 | Trichocomaceae | 26 | Aspergillus | 26 |
| Metazoa | 397 | Basidiomycota | 14 | Leotiomycetes | 1 | Helotiales | 1 | Sclerotiniaceae | 1 | Sclerotinia | 1 |
| Viridiplantae | 274 | Arthropoda | 309 | Saccharomycetes | 7 | Saccharomycetales | 7 | Debaryomycetaceae | 3 | Schwanniomyces | 3 |
|  |  | Chordata | 60 | Schizosaccharomycetes | 5 | Schizosaccharomycetales | 5 | Lipomycetaceae | 1 | Lipomyces | 1 |
|  |  | Echinodermata | 1 | Agaricomycetes | 12 | Cantharellales | 11 | Phaffomycetaceae | 1 | Wickerhamomyces | 1 |
|  |  | Mollusca | 25 | Tremellomycetes | 2 | Sebacinales | 1 | Saccharomycopsidaceae | 2 | Saccharomycopsis | 2 |
|  |  | Nematoda | 2 | Arachnida | 5 | Tremellales | 2 | Schizosaccharomycetaceae | 5 | Schizosaccharomyces | 5 |
|  |  | Chlorophyta | 9 | Branchiopoda | 2 | Astigmata | 3 | Ceratobasidiaceae | 11 | Rhizoctonia | 11 |
|  |  | Streptophyta | 265 | Chilopoda | 2 | Ixodida | 1 | Sebacinaceae | 1 | Piriformospora | 1 |
|  |  |  |  | Insecta | 292 | Scorpiones | 1 | Tremellaceae | 2 | Filobasidiella | 2 |
|  |  |  |  | Malacostraca | 8 | Diplostraca | 2 | Pyroglyphidae | 3 | Dermatophagoides | 2 |
|  |  |  |  | Actinopterygii | 27 | Lithobiomorpha | 2 | Ixodidae | 1 | Euroglyphus | 1 |
|  |  |  |  | Amphibia | 1 | Blattodea | 2 | Buthidae | 1 | Ixodes | 1 |
|  |  |  |  | Aves | 4 | Coleoptera | 17 | Daphniidae | 2 | Tityus | 1 |
|  |  |  |  | Mammalia | 28 | Diptera | 249 | Lithobiidae | 2 | Daphnia | 2 |
|  |  |  |  | Asteroidea | 1 | Hemiptera | 1 | Ectobiidae | 2 | Lithobius | 2 |
|  |  |  |  | Bivalvia | 23 | Hymenoptera | 8 | Chrysomelidae | 5 | Blattella | 2 |
|  |  |  |  | Polyplacophora | 2 | Lepidoptera | 15 | Curculionidae | 6 | Callosobruchus | 1 |
|  |  |  |  | Enoplea | 2 | Decapoda | 8 | Tenebrionidae | 6 | Diabrotica | 2 |
|  |  |  |  | Chlorophyceae | 7 | Anguilliformes | 1 | Bibionidae | 2 | Phaedon | 1 |
|  |  |  |  | Trebouxiophyceae | 2 | Cypriniformes | 4 | Culicidae | 16 | Zabrotes | 1 |
|  |  |  |  | Bryopsida | 4 | Osmeriformes | 1 | Drosophilidae | 221 | Anthonomus | 2 |
|  |  |  |  | Liliopsida | 261 | Perciformes | 14 | Glossinidae | 1 | Ips | 2 |
|  |  |  |  |  |  | Pleuronectiformes | 2 | Muscidae | 1 | Sitophilus | 2 |
|  |  |  |  |  |  | Salmoniformes | 1 | Phoridae | 1 | Blaps | 1 |
|  |  |  |  |  |  | Tetraodontiformes | 4 | Psychodidae | 2 | Tenebrio | 1 |
|  |  |  |  |  |  | Anura | 1 | Simuliidae | 2 | Tribolium | 4 |
|  |  |  |  |  |  | Anseriformes | 1 | Stratiomyidae | 1 | Bibio | 2 |
|  |  |  |  |  |  | Galliformes | 2 | Tephritidae | 2 | Aedes | 2 |
|  |  |  |  |  |  | Struthioniformes | 1 | Reduviidae | 1 | Anopheles | 3 |
|  |  |  |  |  |  | Chiroptera | 2 | Apidae | 2 | Culex | 10 |
|  |  |  |  |  |  | Primates | 17 | Braconidae | 1 | Ochlerotatus | 1 |
|  |  |  |  |  |  | Rodentia | 8 | Formicidae | 5 | Drosophila | 210 |
|  |  |  |  |  |  | Scandentia | 1 | Bombycidae | 1 | Hirtodrosophila | 3 |
|  |  |  |  |  |  | Forcipulatida | 1 | Crambidae | 6 | Liodrosophila | 1 |
|  |  |  |  |  |  | Ostreoida | 15 | Noctuidae | 4 | Samoaia | 1 |
|  |  |  |  |  |  | Pectinoida | 2 | Nymphalidae | 3 | Scaptodrosophila | 3 |
|  |  |  |  |  |  | Pterioida | 2 | Pyralidae | 1 | Zaprionus | 3 |
|  |  |  |  |  |  | Veneroida | 4 | Penaeidae | 8 | Glossina | 1 |
|  |  |  |  |  |  | Neoloricata | 2 | Anguillidae | 1 | Musca | 1 |
|  |  |  |  |  |  | Trichocephalida | 2 | Catostomidae | 1 | Megaselia | 1 |
|  |  |  |  |  |  | Chlamydomonadales | 7 | Cyprinidae | 3 | Lutzomyia | 1 |
|  |  |  |  |  |  | Chlorellales | 2 | Osmeridae | 1 | Phlebotomus | 1 |
|  |  |  |  |  |  | Funariales | 4 | Cichlidae | 1 | Simulium | 2 |
|  |  |  |  |  |  | Poales | 253 | Latidae | 1 | Hermetia | 1 |
|  |  |  |  |  |  | Zingiberales | 8 | Moronidae | 1 | Ceratitis | 2 |
|  |  |  |  |  |  |  |  | Serranidae | 1 | Rhodnius | 1 |
|  |  |  |  |  |  |  |  | Scombridae | 3 | Apis | 2 |
|  |  |  |  |  |  |  |  | Sinipercidae | 2 | Cotesia | 1 |
|  |  |  |  |  |  |  |  | Sparidae | 3 | Acromyrmex | 2 |
|  |  |  |  |  |  |  |  | Stichaeidae | 2 | Camponotus | 2 |
|  |  |  |  |  |  |  |  | Pleuronectidae | 2 | Harpegnathos | 1 |
|  |  |  |  |  |  |  |  | Salmonidae | 1 | Bombyx | 1 |
|  |  |  |  |  |  |  |  | Tetraodontidae | 4 | Diatraea | 3 |
|  |  |  |  |  |  |  |  | Pipidae | 1 | Ostrinia | 2 |
|  |  |  |  |  |  |  |  | Anatidae | 1 | Scirpophaga | 1 |
|  |  |  |  |  |  |  |  | Phasianidae | 2 | Helicoverpa | 2 |
|  |  |  |  |  |  |  |  | Struthionidae | 1 | Mamestra | 1 |
|  |  |  |  |  |  |  |  | Pteropodidae | 1 | Spodoptera | 1 |
|  |  |  |  |  |  |  |  | Vespertilionidae | 1 | Danaus | 3 |
|  |  |  |  |  |  |  |  | Cercopithecidae | 4 | Ephestia | 1 |
|  |  |  |  |  |  |  |  | Hominidae | 13 | Farfantepenaeus | 1 |
|  |  |  |  |  |  |  |  | Bathyergidae | 2 | Litopenaeus | 7 |
|  |  |  |  |  |  |  |  | Cricetidae | 1 | Anguilla | 1 |
|  |  |  |  |  |  |  |  | Muridae | 5 | Myxocyprinus | 1 |
|  |  |  |  |  |  |  |  | Tupaiidae | 1 | Ctenopharyngodon | 1 |
|  |  |  |  |  |  |  |  | Asteriidae | 1 | Danio | 2 |
|  |  |  |  |  |  |  |  | Ostreidae | 15 | Osmerus | 1 |
|  |  |  |  |  |  |  |  | Pectinidae | 1 | Oreochromis | 1 |
|  |  |  |  |  |  |  |  | Spondylidae | 1 | Lates | 1 |
|  |  |  |  |  |  |  |  | Pteriidae | 2 | Dicentrarchus | 1 |
|  |  |  |  |  |  |  |  | Cardiidae | 1 | Epinephelus | 1 |
|  |  |  |  |  |  |  |  | Corbiculidae | 1 | Thunnus | 3 |
|  |  |  |  |  |  |  |  | Dreissenidae | 1 | Siniperca | 2 |
|  |  |  |  |  |  |  |  | Veneridae | 1 | Diplodus | 1 |
|  |  |  |  |  |  |  |  | Acanthochitonidae | 2 | Pagrus | 2 |
|  |  |  |  |  |  |  |  | Trichinellidae | 2 | Xiphister | 2 |
|  |  |  |  |  |  |  |  | Chlamydomonadaceae | 6 | Pseudopleuronectes | 2 |
|  |  |  |  |  |  |  |  | Volvocaceae | 1 | Salmo | 1 |
|  |  |  |  |  |  |  |  | Chlorellaceae | 2 | Tetraodon | 4 |
|  |  |  |  |  |  |  |  | Funariaceae | 4 | Xenopus | 1 |
|  |  |  |  |  |  |  |  | Poaceae | 253 | Anas | 1 |
|  |  |  |  |  |  |  |  | Musaceae | 8 | Gallus | 2 |
|  |  |  |  |  |  |  |  |  |  | Struthio | 1 |
|  |  |  |  |  |  |  |  |  |  | Pteropus | 1 |
|  |  |  |  |  |  |  |  |  |  | Myotis | 1 |
|  |  |  |  |  |  |  |  |  |  | Colobus | 1 |
|  |  |  |  |  |  |  |  |  |  | Macaca | 3 |
|  |  |  |  |  |  |  |  |  |  | Gorilla | 3 |
|  |  |  |  |  |  |  |  |  |  | Homo | 5 |
|  |  |  |  |  |  |  |  |  |  | Pan | 5 |
|  |  |  |  |  |  |  |  |  |  | Heterocephalus | 2 |
|  |  |  |  |  |  |  |  |  |  | Myodes | 1 |
|  |  |  |  |  |  |  |  |  |  | Mus | 2 |
|  |  |  |  |  |  |  |  |  |  | Rattus | 3 |
|  |  |  |  |  |  |  |  |  |  | Tupaia | 1 |
|  |  |  |  |  |  |  |  |  |  | Asterias | 1 |
|  |  |  |  |  |  |  |  |  |  | Crassostrea | 15 |
|  |  |  |  |  |  |  |  |  |  | Pecten | 1 |
|  |  |  |  |  |  |  |  |  |  | Spondylus | 1 |
|  |  |  |  |  |  |  |  |  |  | Pinctada | 1 |
|  |  |  |  |  |  |  |  |  |  | Pteria | 1 |
|  |  |  |  |  |  |  |  |  |  | Cerastoderma | 1 |
|  |  |  |  |  |  |  |  |  |  | Corbicula | 1 |
|  |  |  |  |  |  |  |  |  |  | Dreissena | 1 |
|  |  |  |  |  |  |  |  |  |  | Ruditapes | 1 |
|  |  |  |  |  |  |  |  |  |  | Acanthochitona | 2 |
|  |  |  |  |  |  |  |  |  |  | Trichinella | 2 |
|  |  |  |  |  |  |  |  |  |  | Chlamydomonas | 6 |
|  |  |  |  |  |  |  |  |  |  | Volvox | 1 |
|  |  |  |  |  |  |  |  |  |  | Chlorella | 1 |
|  |  |  |  |  |  |  |  |  |  | Prototheca | 1 |
|  |  |  |  |  |  |  |  |  |  | Physcomitrella | 4 |
|  |  |  |  |  |  |  |  |  |  | Aegilops | 8 |
|  |  |  |  |  |  |  |  |  |  | Agropyron | 1 |
|  |  |  |  |  |  |  |  |  |  | Australopyrum | 2 |
|  |  |  |  |  |  |  |  |  |  | Avena | 2 |
|  |  |  |  |  |  |  |  |  |  | Brachypodium | 4 |
|  |  |  |  |  |  |  |  |  |  | Bromus | 1 |
|  |  |  |  |  |  |  |  |  |  | Crithopsis | 1 |
|  |  |  |  |  |  |  |  |  |  | Dactylis | 2 |
|  |  |  |  |  |  |  |  |  |  | Dasypyrum | 2 |
|  |  |  |  |  |  |  |  |  |  | Elymus | 82 |
|  |  |  |  |  |  |  |  |  |  | Eremopyrum | 3 |
|  |  |  |  |  |  |  |  |  |  | Henrardia | 1 |
|  |  |  |  |  |  |  |  |  |  | Heteranthelium | 1 |
|  |  |  |  |  |  |  |  |  |  | Hordeum | 70 |
|  |  |  |  |  |  |  |  |  |  | Lophopyrum | 1 |
|  |  |  |  |  |  |  |  |  |  | Oryza | 34 |
|  |  |  |  |  |  |  |  |  |  | Peridictyon | 1 |
|  |  |  |  |  |  |  |  |  |  | Psathyrostachys | 2 |
|  |  |  |  |  |  |  |  |  |  | Secale | 6 |
|  |  |  |  |  |  |  |  |  |  | Setaria | 4 |
|  |  |  |  |  |  |  |  |  |  | Sorghum | 4 |
|  |  |  |  |  |  |  |  |  |  | Taeniatherum | 4 |
|  |  |  |  |  |  |  |  |  |  | Thinopyrum | 2 |
|  |  |  |  |  |  |  |  |  |  | Triticum | 7 |
|  |  |  |  |  |  |  |  |  |  | x Triticosecale | 1 |
|  |  |  |  |  |  |  |  |  |  | Zea | 7 |
|  |  |  |  |  |  |  |  |  |  | Musa | 8 |

Table S4 Taxonomic classification of 2586 amylases from bacteria used in this study

| Phylum |  | Class |  | Order |  | Family |  | Genus |  |
| --- | --- | --- | --- | --- | --- | --- | --- | --- | --- |
| Armatimonadetes | 2 | Chthonomonadetes | 2 | Chthonomonadales | 2 | Chthonomonadaceae | 2 | Chthonomonas | 2 |
| Bacteroidetes | 104 | Bacteroidia | 58 | Bacteroidales | 58 | Bacteroidaceae | 12 | Bacteroides | 12 |
| Chlorobi | 1 | Cytophagia | 19 | Cytophagales | 19 | Porphyromonadaceae | 5 | Odoribacter | 1 |
| Chloroflexi | 1 | Flavobacteriia | 25 | Flavobacteriales | 25 | Prevotellaceae | 39 | Parabacteroides | 4 |
| Deinococcus-Thermus | 1 | Sphingobacteriia | 2 | Sphingobacteriales | 2 | Rikenellaceae | 2 | Prevotella | 39 |
| Firmicutes | 698 | Chlorobia | 1 | Chlorobiales | 1 | Cyclobacteriaceae | 4 | Alistipes | 2 |
| Planctomycetes | 4 | Ktedonobacteria | 1 | Ktedonobacterales | 1 | Cytophagaceae | 9 | Cecembia | 3 |
| Proteobacteria | 1770 | Deinococci | 1 | Thermales | 1 | Flammeovirgaceae | 6 | Nitritalea | 1 |
| Tenericutes | 5 | Bacilli | 618 | Bacillales | 147 | Flavobacteriaceae | 25 | Cytophaga | 2 |
|  |  | Clostridia | 78 | Lactobacillales | 471 | Sphingobacteriaceae | 2 | Fibrella | 2 |
|  |  | Negativicutes | 2 | Clostridiales | 71 | Chlorobiaceae | 1 | Fibrisoma | 4 |
|  |  | Phycisphaerae | 1 | Halanaerobiales | 3 | Ktedonobacteraceae | 1 | Microscilla | 1 |
|  |  | Planctomycetia | 3 | Thermoanaerobacterales | 4 | Thermaceae | 1 | Cesiribacter | 6 |
|  |  | Alphaproteobacteria | 9 | Selenomonadales | 2 | Bacillaceae | 134 | Capnocytophaga | 6 |
|  |  | Betaproteobacteria | 17 | Phycisphaerales | 1 | Paenibacillaceae | 10 | Chryseobacterium | 3 |
|  |  | Deltaproteobacteria | 18 | Planctomycetales | 3 | Planococcaceae | 2 | Dokdonia | 1 |
|  |  | Gammaproteobacteria | 1726 | Caulobacterales | 1 | Thermoactinomycetaceae | 1 | Flavobacterium | 6 |
|  |  | Mollicutes | 5 | Rhizobiales | 4 | Aerococcaceae | 4 | Galbibacter | 1 |
|  |  |  |  | Rhodobacterales | 2 | Carnobacteriaceae | 2 | Gramella | 3 |
|  |  |  |  | Rhodospirillales | 2 | Enterococcaceae | 12 | Kordia | 1 |
|  |  |  |  | Burkholderiales | 12 | Lactobacillaceae | 26 | Nonlabens | 2 |
|  |  |  |  | Methylophilales | 1 | Streptococcaceae | 427 | Zobellia | 2 |
|  |  |  |  | Nitrosomonadales | 2 | Clostridiaceae | 35 | Arcticibacter | 1 |
|  |  |  |  | Rhodocyclales | 2 | Clostridiales Family XI Incertae Sedis | 6 | Pedobacter | 1 |
|  |  |  |  | Bdellovibrionales | 2 | Eubacteriaceae | 17 | Chlorobaculum | 1 |
|  |  |  |  | Desulfobacterales | 1 | Lachnospiraceae | 12 | Ktedonobacter | 1 |
|  |  |  |  | Desulfovibrionales | 3 | Ruminococcaceae | 1 | Meiothermus | 1 |
|  |  |  |  | Myxococcales | 8 | Halanaerobiaceae | 3 | Anoxybacillus | 2 |
|  |  |  |  | Syntrophobacterales | 4 | Thermoanaerobacteraceae | 1 | Bacillus | 116 |
|  |  |  |  | Aeromonadales | 5 | Thermoanaerobacterales Family III Incertae Sedis | 1 | Geobacillus | 12 |
|  |  |  |  | Alteromonadales | 23 | Thermodesulfobiaceae | 2 | Halobacillus | 1 |
|  |  |  |  | Chromatiales | 2 | Veillonellaceae | 2 | Lysinibacillus | 2 |
|  |  |  |  | Enterobacteriales | 1360 | Phycisphaeraceae | 1 | Oceanobacillus | 1 |
|  |  |  |  | Legionellales | 8 | Planctomycetaceae | 3 | Paenibacillus | 10 |
|  |  |  |  | Methylococcales | 3 | Caulobacteraceae | 1 | Sporosarcina | 2 |
|  |  |  |  | Pasteurellales | 6 | Rhizobiaceae | 4 | Thermoactinomyces | 1 |
|  |  |  |  | Pseudomonadales | 26 | Rhodobacteraceae | 2 | Aerococcus | 4 |
|  |  |  |  | Vibrionales | 289 | Rhodospirillaceae | 2 | Carnobacterium | 2 |
|  |  |  |  | Xanthomonadales | 4 | Burkholderiaceae | 10 | Enterococcus | 9 |
|  |  |  |  | Acholeplasmatales | 4 | Oxalobacteraceae | 2 | Melissococcus | 2 |
|  |  |  |  | Entomoplasmatales | 1 | Methylophilaceae | 1 | Tetragenococcus | 1 |
|  |  |  |  |  |  | Nitrosomonadaceae | 2 | Lactobacillus | 26 |
|  |  |  |  |  |  | Rhodocyclaceae | 2 | Lactococcus | 9 |
|  |  |  |  |  |  | Bacteriovoracaceae | 1 | Streptococcus | 418 |
|  |  |  |  |  |  | Bdellovibrionaceae | 1 | Caloramator | 2 |
|  |  |  |  |  |  | Desulfobacteraceae | 1 | Clostridium | 31 |
|  |  |  |  |  |  | Desulfovibrionaceae | 3 | Thermobrachium | 2 |
|  |  |  |  |  |  | Cystobacteraceae | 5 | Anaerococcus | 2 |
|  |  |  |  |  |  | Kofleriaceae | 1 | Finegoldia | 4 |
|  |  |  |  |  |  | Myxococcaceae | 1 | Eubacterium | 16 |
|  |  |  |  |  |  | Polyangiaceae | 1 | Pseudoramibacter | 1 |
|  |  |  |  |  |  | Syntrophaceae | 4 | Butyrivibrio | 5 |
|  |  |  |  |  |  | Aeromonadaceae | 5 | Cellulosilyticum | 1 |
|  |  |  |  |  |  | Alteromonadaceae | 12 | Lachnoanaerobaculum | 4 |
|  |  |  |  |  |  | Colwelliaceae | 1 | Roseburia | 2 |
|  |  |  |  |  |  | Pseudoalteromonadaceae | 7 | Ruminococcus | 1 |
|  |  |  |  |  |  | Shewanellaceae | 3 | Halanaerobium | 1 |
|  |  |  |  |  |  | Chromatiaceae | 2 | Halothermothrix | 2 |
|  |  |  |  |  |  | Enterobacteriaceae | 1360 | Caldanaerobacter | 1 |
|  |  |  |  |  |  | Legionellaceae | 8 | Thermoanaerobacterium | 1 |
|  |  |  |  |  |  | Methylococcaceae | 3 | Coprothermobacter | 2 |
|  |  |  |  |  |  | Pasteurellaceae | 6 | Selenomonas | 2 |
|  |  |  |  |  |  | Pseudomonadaceae | 26 | Phycisphaera | 1 |
|  |  |  |  |  |  | Vibrionaceae | 289 | Planctomyces | 1 |
|  |  |  |  |  |  | Xanthomonadaceae | 4 | Rhodopirellula | 2 |
|  |  |  |  |  |  | Acholeplasmataceae | 4 | Caulobacter | 1 |
|  |  |  |  |  |  | Spiroplasmataceae | 1 | Agrobacterium | 3 |
|  |  |  |  |  |  |  |  | Rhizobium | 1 |
|  |  |  |  |  |  |  |  | Dinoroseobacter | 1 |
|  |  |  |  |  |  |  |  | Ketogulonicigenium | 1 |
|  |  |  |  |  |  |  |  | Magnetospirillum | 1 |
|  |  |  |  |  |  |  |  | Rhodospirillum | 1 |
|  |  |  |  |  |  |  |  | Burkholderia | 4 |
|  |  |  |  |  |  |  |  | Lautropia | 2 |
|  |  |  |  |  |  |  |  | Ralstonia | 4 |
|  |  |  |  |  |  |  |  | Janthinobacterium | 2 |
|  |  |  |  |  |  |  |  | Methylobacillus | 1 |
|  |  |  |  |  |  |  |  | Nitrosomonas | 1 |
|  |  |  |  |  |  |  |  | Nitrosospira | 1 |
|  |  |  |  |  |  |  |  | Aromatoleum | 1 |
|  |  |  |  |  |  |  |  | Dechloromonas | 1 |
|  |  |  |  |  |  |  |  | Bacteriovorax | 1 |
|  |  |  |  |  |  |  |  | Bdellovibrio | 1 |
|  |  |  |  |  |  |  |  | Desulfotignum | 1 |
|  |  |  |  |  |  |  |  | Desulfovibrio | 3 |
|  |  |  |  |  |  |  |  | Stigmatella | 5 |
|  |  |  |  |  |  |  |  | Haliangium | 1 |
|  |  |  |  |  |  |  |  | Myxococcus | 1 |
|  |  |  |  |  |  |  |  | Sorangium | 1 |
|  |  |  |  |  |  |  |  | Syntrophus | 4 |
|  |  |  |  |  |  |  |  | Aeromonas | 5 |
|  |  |  |  |  |  |  |  | Agarivorans | 6 |
|  |  |  |  |  |  |  |  | Alishewanella | 2 |
|  |  |  |  |  |  |  |  | Glaciecola | 4 |
|  |  |  |  |  |  |  |  | Colwellia | 1 |
|  |  |  |  |  |  |  |  | Pseudoalteromonas | 7 |
|  |  |  |  |  |  |  |  | Shewanella | 3 |
|  |  |  |  |  |  |  |  | Rheinheimera | 2 |
|  |  |  |  |  |  |  |  | Citrobacter | 9 |
|  |  |  |  |  |  |  |  | Cronobacter | 14 |
|  |  |  |  |  |  |  |  | Edwardsiella | 3 |
|  |  |  |  |  |  |  |  | Enterobacter | 11 |
|  |  |  |  |  |  |  |  | Erwinia | 17 |
|  |  |  |  |  |  |  |  | Escherichia | 826 |
|  |  |  |  |  |  |  |  | Klebsiella | 58 |
|  |  |  |  |  |  |  |  | Kosakonia | 1 |
|  |  |  |  |  |  |  |  | Pantoea | 3 |
|  |  |  |  |  |  |  |  | Rahnella | 1 |
|  |  |  |  |  |  |  |  | Raoultella | 1 |
|  |  |  |  |  |  |  |  | Salmonella | 262 |
|  |  |  |  |  |  |  |  | Serratia | 10 |
|  |  |  |  |  |  |  |  | Shigella | 60 |
|  |  |  |  |  |  |  |  | Yersinia | 84 |
|  |  |  |  |  |  |  |  | Legionella | 8 |
|  |  |  |  |  |  |  |  | Methylomicrobium | 3 |
|  |  |  |  |  |  |  |  | Actinobacillus | 1 |
|  |  |  |  |  |  |  |  | Aggregatibacter | 2 |
|  |  |  |  |  |  |  |  | Mannheimia | 2 |
|  |  |  |  |  |  |  |  | Pasteurella | 1 |
|  |  |  |  |  |  |  |  | Cellvibrio | 11 |
|  |  |  |  |  |  |  |  | Pseudomonas | 15 |
|  |  |  |  |  |  |  |  | Aliivibrio | 5 |
|  |  |  |  |  |  |  |  | Photobacterium | 1 |
|  |  |  |  |  |  |  |  | Vibrio | 283 |
|  |  |  |  |  |  |  |  | Stenotrophomonas | 1 |
|  |  |  |  |  |  |  |  | Xanthomonas | 3 |
|  |  |  |  |  |  |  |  | Acholeplasma | 4 |
|  |  |  |  |  |  |  |  | Spiroplasma | 1 |
